# The graphical brain: Belief propagation and active inference

**DOI:** 10.1162/NETN_a_00018

**Published:** 2017-12-31

**Authors:** Karl J. Friston, Thomas Parr, Bert de Vries

**Affiliations:** 1 Wellcome Trust Centre for Neuroimaging, Institute of Neurology, University College London, United Kingdom; 2 Eindhoven University of Technology, Department of Electrical Engineering, Eindhoven, The Netherlands; 3 GN Hearing, Eindhoven, The Netherlands

**Keywords:** Bayesian, Neuronal, Connectivity, Factor graphs, Free energy, Belief propagation, Message passing

## Abstract

This paper considers functional integration in the brain from a computational perspective. We ask what sort of neuronal message passing is mandated by active inference—and what implications this has for context-sensitive connectivity at microscopic and macroscopic levels. In particular, we formulate neuronal processing as belief propagation under deep generative models. Crucially, these models can entertain both discrete and continuous states, leading to distinct schemes for belief updating that play out on the same (neuronal) architecture. Technically, we use Forney (normal) factor graphs to elucidate the requisite message passing in terms of its form and scheduling. To accommodate mixed generative models (of discrete and continuous states), one also has to consider link nodes or factors that enable discrete and continuous representations to talk to each other. When mapping the implicit computational architecture onto neuronal connectivity, several interesting features emerge. For example, Bayesian model averaging and comparison, which link discrete and continuous states, may be implemented in thalamocortical loops. These and other considerations speak to a computational connectome that is inherently state dependent and self-organizing in ways that yield to a principled (variational) account. We conclude with simulations of reading that illustrate the implicit neuronal message passing, with a special focus on how discrete (semantic) representations inform, and are informed by, continuous (visual) sampling of the sensorium.

**Author Summary:**

This paper considers functional integration in the brain from a computational perspective. We ask what sort of neuronal message passing is mandated by active inference—and what implications this has for context-sensitive connectivity at microscopic and macroscopic levels. In particular, we formulate neuronal processing as belief propagation under deep generative models that can entertain both discrete and continuous states. This leads to distinct schemes for belief updating that play out on the same (neuronal) architecture. Technically, we use Forney (normal) factor graphs to characterize the requisite message passing, and link this formal characterization to canonical microcircuits and extrinsic connectivity in the brain.

## Introduction

This paper attempts to describe functional integration in the brain in terms of neuronal computations. We start by asking what the brain does, to see how far the implicit constraints on neuronal message passing can take us. In particular, we assume that the brain engages in some form of (Bayesian) inference—and can therefore be described as maximizing Bayesian model evidence (Clark, 2013; Friston, Kilner, & Harrison, 2006; Hohwy, 2016; Mumford, 1992). This implies that the brain embodies a generative model, for which it tries to gather the greatest evidence. On this view, to understand functional integration is to understand the form of the generative model and how it is used to make inferences about sensory data that are sampled actively from the world. Happily, there is an enormous amount known about the various schemes that can implement this form of (Bayesian) inference, thereby offering the possibility of developing a process theory (i.e., neuronally plausible scheme) that implements the normative principle of self-evidencing (Hohwy, 2016).

In brief, this (rather long) paper tries to integrate three themes to provide a rounded perspective on message passing or belief propagation in the brain. These themes include (a) the formal basis of belief propagation, from the perspective of the Bayesian brain and active inference; (b) the biological substrates of the implicit message passing; and (c) how discrete representations (e.g., semantics) might talk to representations of continuous quantities (e.g., visual contrast luminance). Technically, the key contributions are twofold: first, the derivation of belief propagation and Bayesian filtering in generalized coordinates of motion, under the framework afforded by factor graphs. This derivation highlights the similarities between representations of trajectories over future time points, in discrete models, and the representation of trajectories in generalized coordinates of motion in continuous models. Second, we described a fairly generic way in which discrete and continuous representations can be linked through Bayesian model selection and averaging. To leverage these technical developments, for an understanding of brain function, we highlight the constraints they offer on the structure and dynamics of neuronal message passing, using coarse-grained evidence from anatomy and neurophysiology. Finally, the nature of this message passing is illustrated using simulations of pictographic reading.

In what follows, we use graphical representations to characterize message passing under deep (hierarchical) generative models that might be used by the brain. We use three sorts of graphs to emphasize the form of generative models, the nature of Bayesian belief updating, and how this might be accomplished in neuronal circuits—both at the level of macroscopic cortical hierarchies and at the more detailed level of canonical microcircuits. The three sorts of graphs include *Bayesian networks* or dependency graphs (MacKay, 1995; Pearl, 1988), where nodes correspond to unknown variables that have to be inferred and the edges denote dependencies among these (random) variables. This provides a concise description of how (e.g., sensory) data are generated. To highlight the requisite message passing and computational architecture, we will use Forney or normal style *factor graphs* (Loeliger, 2002). In Forney factor graphs, the nodes now represent local functions or factors of a probability distribution over the random variables, while edges come to represent the variables per se (or more exactly a probability distribution over those variables). Finally, we will use *neural networks* or circuits where the nodes are constituted by the sufficient statistics of unknown variables and other auxiliary variables, such as prediction errors. The edges in these graphs denote an exchange of (functions of) sufficient statistics—of the sort one can interpret in terms of neuronal (axonal) connections. Crucially, these graphical representations are formally equivalent in the sense that any Bayesian network can be expressed as a factor graph. And any message passing on a factor graph can be depicted as a neural network. However, as we will see later, the various graphical formulations offer different perspectives on belief updating or propagation. We will leverage these perspectives to work from a purely normative theory (based upon the maximization of Bayesian model evidence or minimization of variational free energy) towards a process theory (based upon belief propagation and the attractors of dynamical systems).

In this paper, we use Forney factor graphs for purely didactic purposes; namely, to illustrate the simplicity with which messages are composed in belief propagation—and to emphasize the recurrent aspect of message passing. However, articulating a generative model as a Forney factor graph has many practical advantages, especially in a computer science or implementational setting. Factor graphs are an important type of probabilistic graphical model because they facilitate the derivation of (approximate) Bayesian inference algorithms. When a generative model is specified as a factor graph, latent variables can often be inferred by executing a message passing schedule that can be derived automatically. Examples include the sumproduct algorithm (belief propagation) for exact inference, and variational message passing and expectation propagation (EP) for approximate inference (Dauwels, 2007). Probabilistic (*hybrid* or mixed) models (Buss, 2003) that include both continuous and discrete variables require a link factor, such as the logistic or probit link function. We will use a generic link factor that implements post hoc Bayesian model comparison and averaging (K. Friston & Penny, 2011; Hoeting, Madigan, Raftery, & Volinsky, 1999). Technically, equipping generative models of latent categorical states with the ability to handle continuous data means that one can categorize continuous data—and use posterior beliefs about categories as empirical priors for processing continuous data (e.g., time series). Clearly, this is something that the brain does all the time during perceptual categorization and action selection. For an introduction to Forney factor graphs, see Kschischang, Frey, and Loeliger (2001) and Loeliger (2002).

This paper comprises six sections. The first overviews active inference in terms of the (normative) imperative to minimize surprise, resolve uncertainty, and (implicitly) maximize model evidence. This section focuses on the selection of actions or policies (sequences of actions) that minimize expected free energy—and what this entails intuitively. Having established the basic premise that the brain engages in active (Bayesian) inference, we then turn to the generative models for which evidence is sought. The second section considers models where the states or causes of data are discrete or categorical in nature. In particular, it considers generative models based upon Markov decision processes, characterized in terms of Bayesian networks and Forney factor graphs. From these, we develop a putative (neural network) microcircuitry that could implement the requisite belief propagation. This section also takes the opportunity to distinguish between *Bayesian* inference and *active* inference by combining (Bayesian network and Forney factor) graphical formulations to show how the products of inference couple back to the process of generating (sensory) data, thereby enabling the brain to author the data or evidence that it accumulates. This section concludes by describing deep models that are composed of Markov decision processes, nested hierarchically over time.

The third section applies the same treatment to generative models with continuous states using a general formulation, based on generalized coordinates of motion (K. Friston, Stephan, Li, & Daunizeau, 2010). This treatment emphasizes the formal equivalence with belief propagation under discrete models. The fourth section considers generative models in which a Markov decision process is placed on top of a continuous state space model. This section deals with the special problem of how the two parts of the model are linked. The technical contribution of this paper is to link continuous states to discrete states through Bayesian model averages of discrete priors (over the causes of continuous dynamics). Conversely, the posterior probability density over these causes is converted into a discrete representation through Bayesian model comparison. The section concludes with a proposal for extrinsic (between-region) message passing in the brain that is consistent with the architecture of belief propagation under mixed generative models. In particular, we highlight the possible role of message passing between cortical and subcortical (basal ganglia and thalamic) systems. The fifth section illustrates belief propagation and the attendant process theory using simulations of (metaphorical) reading. This section shows how message passing works and clarifies notions like hidden states, using letters, words, and sentences. Crucially, inferences made about (discrete) words and sentences use (continuous) sensory data solicited by saccadic eye movements that accrue visual (and proprioceptive) input over time. We conclude with a brief section on the implications for context-sensitive connectivity that can be induced under the belief propagation scheme. We focus on the modulation of intrinsic excitability; specifically, the afferents to superficial pyramidal cells—and, as a more abstract level, the implications for self-organized criticality of the sort entailed by dynamic fluctuations in connectivity (Aertsen, Gerstein, Habib, & Palm, 1989; Allen et al., 2012; Baker et al., 2014; Breakspear, 2004).

## Active Inference: Self-Evidencing and Expected Free Energy

All that follows is predicated on defining what the brain does or, more exactly, what properties it must possess to endure in a changing world. In this sense, the brain conforms to the imperatives for all sentient creatures; namely, to restrict itself to a limited number of attracting states (Friston, 2013). Mathematically, this can be cast as minimizing self-information or surprise (in information theoretic terms). Alternatively, this is equivalent to maximizing Bayesian model evidence; namely, the probability of sensory exchanges with the environment, under a model of how those sensations were caused. This is the essence of the free energy principle and its corollary—active inference—that can be neatly summarized as *self-evidencing* (Hohwy, 2016). Intuitively, self-evidencing means the brain can be described as inferring the causes of sensory samples while, at the same time, soliciting sensations that are the least surprising (e.g., not looking at the sun directly or maintaining thermoreceptor firing within a physiological range). Technically, this take on action and perception can be cast as minimizing a proxy for surprise; namely *variational free energy*. Crucially, active inference generalizes Bayesian inference in that the objective is not just to infer the latent or hidden states that cause sensations but to act in a way that will minimize *expected* surprise in the future. In information theory, expected surprise is known as entropy or uncertainty. This means that one can define optimal behavior as acting to resolve uncertainty (e.g., saccading to salient, or information rich, regimes of visual space or avoiding outcomes that are, a priori, costly or unattractive). In the same way that direct action and perception minimize surprise vicariously, through minimizing free energy, action can be specified in terms of policies that minimize the free energy expected when pursuing that policy.

### Expected Free Energy

Expected free energy has a relatively simple form (see Supplementary Information: Friston, Parr, & de Vries, 2017), which can be decomposed into an epistemic, information seeking, uncertainty reducing part (*intrinsic value*) and a pragmatic, goal seeking, cost aversive part (*extrinsic value*). Formally, the expected free energy for a particular policy *π* at time *τ* in the future can be expressed in terms of probabilistic beliefs *Q*(*s_τ_,o_τ_*|*π*) about future states *s_τ_* and outcomes *o_τ_* (see Supplementary Information, Table 1 and Appendix 2: Friston, Parr, et al., 2017):

(1) $$G\left(\pi ,\tau \right)=-\underset{\text{intrinsic}\;\text{value}}{\underset{︸}{E\left[\text{ln}\;Q\left(s_{\tau }|o_{\tau },\pi \right)-\text{ln}\;Q\left(s_{\tau }|\pi \right)\right]}}-\underset{\text{extrinsic}\;\text{value}.}{\underset{︸}{E\left[\text{ln}\;Q\left(o_{\tau }\right)\right]}}$$

Extrinsic (pragmatic) value is simply the expected value of a policy defined in terms of outcomes that are preferred a priori, where the equivalent cost corresponds to prior surprise (see glossary of terms in Table 1–Friston, Parr, et al., 2017). The more interesting part is the uncertainty resolving or intrinsic (epistemic) value, variously referred to as relative entropy, mutual information, information gain, Bayesian surprise, or value of information expected under a particular policy (Barlow, 1961; Howard, 1966; Itti & Baldi, 2009; Linsker, 1990; Optican & Richmond, 1987). An alternative formulation of expected free energy can be found in the Supplementary Information, Appendix 1 (Friston, Parr, et al., 2017), which shows that expected free energy is also the expected uncertainty about outcomes (i.e., *ambiguity*) plus the Kullback-Leibler divergence (i.e., relative entropy or *risk*) between predicted and preferred outcomes. This means that minimizing expected free energy is guaranteed to realize preferred outcomes, while resolving uncertainty about the states of the world generating those outcomes.

In what follows, we will be less concerned with the pragmatic or utilitarian aspect of expected free energy and focus on the epistemic drive to explore salient regimes of the sensorium. We have previously addressed this epistemic foraging in terms of saccadic eye movements, using a generalized Bayesian filter as a model of neuronal dynamics (Friston, Adams, Perrinet, & Breakspear, 2012). In this paper, we reproduce the same sort of behavior but much more efficiently, using a generative model that entertains both discrete and continuous states. In brief, we will use a discrete state space model to generate empirical priors or predictions about where to look next, and a continuous state space model to implement those predictions, thereby garnering (visual) information that enables a constructivist explanation for visual samples: namely scene construction (Hassabis & Maguire, 2007; Mirza, Adams, Mathys, & Friston, 2016). The penultimate section presents simulations of reading to illustrate the use of deep generative models in active inference. However, first we consider the nature of generative models and the belief updating that they entail. In what follows, it will be useful to keep in mind the distinction between a true *generative process* in the real world and an agent’s *generative model* of that process. This distinction is important because active inference deals with how the generative model of a process and the process per se are coupled in a circular fashion to describe the perception-action cycle (Fuster, 2004; Tishby & Polani, 2010).

## Discrete Generative Models

This section focuses on generative models of discrete outcomes caused by discrete states that cannot be observed directly (i.e., latent or hidden states). In brief, the unknown variables in these models correspond to states of the world that generate the outcomes of policies or sequences of actions. Note that policies have to be inferred. In other words, in active inference one has to infer what policy one is currently pursuing, where this inference can be biased by prior beliefs or preferences. It is these prior preferences that lend action a purposeful and goal-directed aspect.

**Figure 1:**
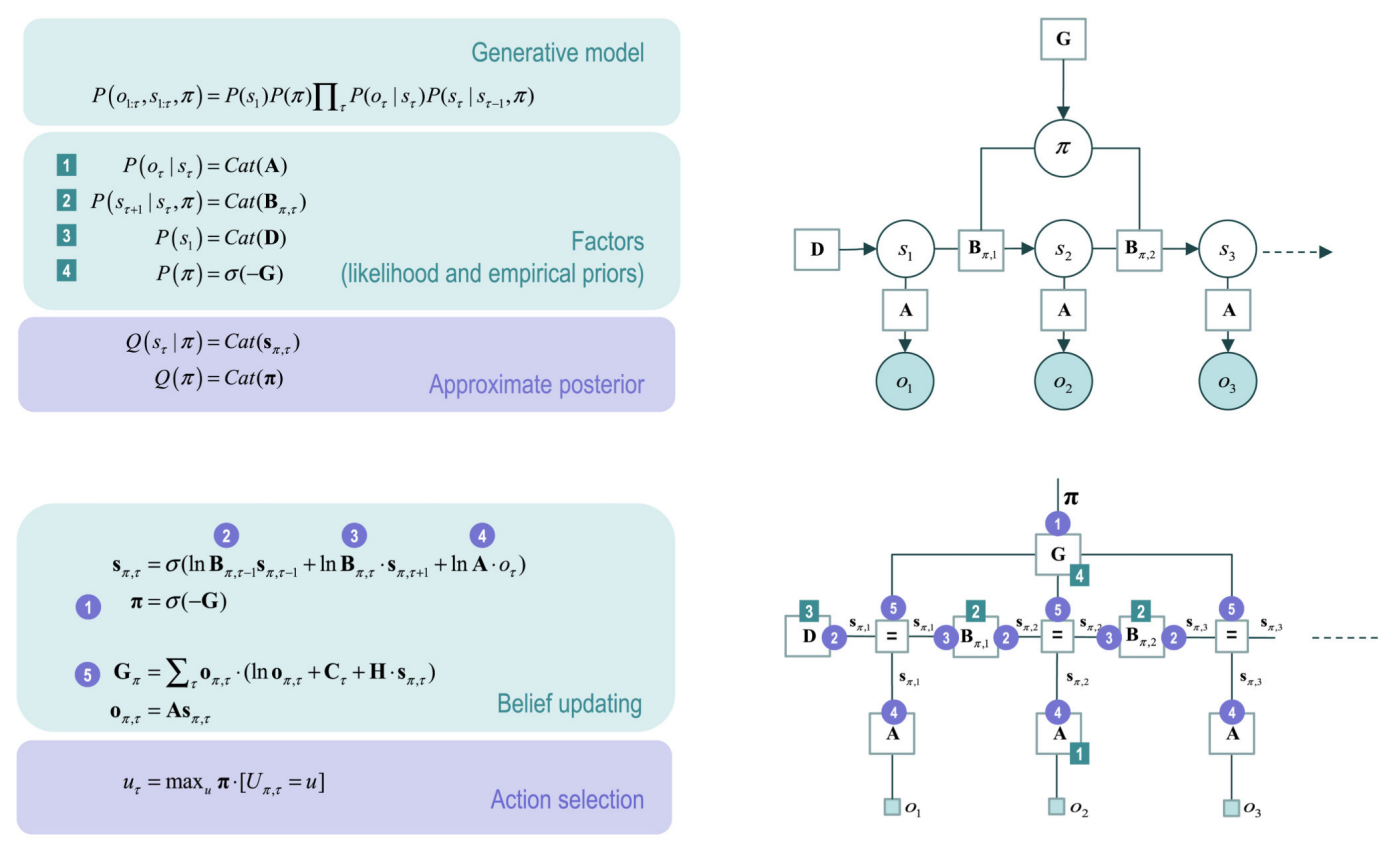
Generative model for discrete states and outcomes. **Upper left panel:** These equations specify the generative model. A generative model is the joint probability of outcomes or consequences and their (latent or hidden) causes; see first equation. Usually, the model is expressed in terms of a *likelihood* (the probability of consequences given causes) and *priors* over causes. When a prior depends upon a random variable it is called an *empirical prior*. Here, the likelihood is specified by a matrix **A** whose elements are the probability of an outcome under every combination of hidden states. *Cat* denotes a categorical probability distribution. The empirical priors pertain to probabilistic transitions (in the **B** matrix) among hidden states that can depend upon actions, which are determined by policies (sequences of actions encoded by *π*). The key aspect of this generative model is that policies are more probable a priori if they minimize the (time integral of) expected free energy **G,** which depends upon prior preferences about outcomes or *costs* encoded in **C** and the uncertainty or ambiguity about outcomes under each state, encoded by **H**. Finally, the vector **D** specifies the initial state. This completes the specification of the model in terms of parameters that constitute **A**, **B**, **C,** and **D**. Bayesian model inversion refers to the inverse mapping from consequences to causes; that is, estimating the hidden states and other variables that cause outcomes. In approximate Bayesian inference, one specifies the form of an approximate posterior distribution. This particular form in this paper uses a mean field approximation, in which posterior beliefs are approximated by the product of marginal distributions over time points. Subscripts index time (or policy). See the main text and Table 1a in Friston, Parr, et al. (2017) for a detailed explanation of the variables (italic variables represent hidden states, while bold variables indicate expectations about those states). Upper right panel: This Bayesian network represents the conditional dependencies among hidden states and how they cause outcomes. Open circles are random variables (hidden states and policies), while filled circles denote observable outcomes. Squares indicate fixed or known variables, such as the model parameters. We have used a slightly unusual convention where parameters have been placed on top of the edges (conditional dependencies) that may mediate. **Lower left panel:** These equalities are the belief updates mediating approximate Bayesian inference and action selection. The (Iverson) brackets in the action selection panel return one if the condition in square brackets is satisfied and zero otherwise. **Lower right panel:** This is an equivalent representation of the Bayesian network in terms of a Forney or normal style factor graph. Here the nodes (square boxes) correspond to factors and the edges are associated with unknown variables. Filled squares denote observable outcomes. The edges are labeled in terms of the sufficient statistics of their marginal posteriors (see approximate posterior). Factors have been labeled intuitively in terms of the parameters encoding the associated probability distributions (on the upper left). The circled numbers correspond to the messages that are passed from nodes to edges (the labels are placed on the edge that carries the message from each node). These correspond to the messages implicit in the belief updates (on the lower left).

Figure 1 describes the basic form of these generative models in complementary formats, and the implicit Bayesian belief updating following the observation of new (sensory) outcomes. The equations on the left specify the generative model in terms of a probability distribution over outcomes, states, and policies that can be expressed in terms of marginal densities or factors. These factors are conditional distributions that entail conditional dependencies, encoded by the edges in the Bayesian network on the upper right. The model in Figure 1 generates outcomes in the following way. First, a policy (i.e., action sequence) is selected at the highest level using a softmax function of the free energy expected under plausible policies. Sequences of hidden states are then generated using the probability transitions specified by the selected policy, which are encoded in **B** matrices. These encode probability transitions in terms of policy-specific categorical distributions. As the policy unfolds, the states generate probabilistic outcomes at each point in time. The likelihood of each outcome is encoded by **A** matrices, in terms of categorical distributions over outcomes, under each state.

The equivalent representation of this graphical model is shown as a Forney factor graph on the lower right. Here, the factors of the generative model (numbers in square boxes) now constitute the nodes and the (probability distribution over the) unknown states are associated with edges. The rules used to construct a factor graph are simple: The edge associated with each variable is connected to the factors in which it participates. If a variable appears in only one factor (e.g., policies), then the edge becomes a half-edge. If a variable appears in more than two factors (e.g., hidden states), then (copies of) the variable are associated with several edges that converge on a special node (labeled with “=”). Known or observed variables are usually denoted with small field squares. Note the formal similarity between the Bayesian network and the Forney factor graph; however, also note the differences. In addition to the movement of random variables to the edges, the edges are undirected in the Forney factor graph. This reflects the fact that messages are sent over edges in both directions. In this sense, the Forney factor graph provides a concise summary of the message passing implicit in Bayesian inference.

Heuristically, to perform inference on these graphs, one clamps the outputs to a particular (observed) value and passes messages from each node to each edge until (if necessary) convergence. The messages from node *N* to edge *V*, usually denoted by *μ_N→V_*, comprise the sufficient statistics of the marginal probability distribution over the edge’s variable. These sufficient statistics (e.g., expectations) encode the requisite posterior probability. To illustrate the composition of messages during belief updating, we will illustrate the derivation of the first update (on the lower left of Figure 1) for expectations about hidden states.

The key aspect of this graph is that it discloses the messages that contribute to the posterior marginal over hidden states; here, conditioned on each policy. These constitute (*forward*: 2) messages from representations of the past, (*backward*: 3) messages from the future, and (*likelihood*: 4) messages from the outcome. Crucially, the past and future are represented at all times so that as new outcomes become available, with passage of time, more likelihood messages participate in the message passing, thereby providing more informed (approximate) posteriors. This effectively performs online data assimilation (mediated by forwarding messages) that is informed by prior beliefs concerning future outcomes (mediated by backward messages). Note that the policy is associated with a half-edge. This is because it appears in only one factor; namely, the probability distribution over policies based upon expected free energy **G**. Furthermore, the policy does not appear to participate in the message passing; however, we will see below that policy expectations play a key role, when we couple the message passing to the generative process—to complete an active inference scheme (and later when we consider the coupling between levels in hierarchical models). The reason the policy is not required for belief propagation among hidden state factors is that we have lumped together the hidden states **s** under each policy as a single variable (and the associated probability factors **B**) for clarity. This means that the message passing among the factors encoding hidden states proceeds in parallel for each policy, irrespective of how likely that policy is. Finally, note that the outcomes that inform the expected free energy are not the observed outcomes but predicted outcomes based upon expected states, under each policy (i.e., message 5).

### Belief Updating and Propagation

Expressing the generative model as a factor graph enables one to see clearly the message passing or belief propagation entailed by inference. For example, the marginal posterior over hidden states at any point in time is, by applying the sum-product rule, the product of all incoming messages to the associated factor node, where (ignoring constants)

(2) $$\begin{array}{l} \;Q\left(s_{\tau }|\pi \right)\propto \mu_{{\text{B}}_{\pi ,\tau -1}\to s_{\tau }|\pi }\times \mu_{{\text{B}}_{\pi ,\tau }\to s_{\tau }|\pi }\times \mu_{\text{A}\to s_{\tau }|\pi }\Rightarrow \\ \text{ln}\;Q\left(s_{\tau }|\pi \right)=\underset{forward\;\left(2\right)}{\underset{︸}{\text{ln}\;\mu_{{\text{B}}_{\pi ,\tau -1}\to s_{\tau }|\pi }}}+\underset{backward\;\left(3\right)}{\underset{︸}{\text{ln}\;\mu_{{\text{B}}_{\pi ,\tau }\to s_{\tau }|\pi }}}+\underset{likelihood\;\left(4\right)}{\underset{︸}{\text{ln}\;\mu_{\text{A}\to s_{\tau }|\pi }}}. \end{array}$$

These correspond to the messages encoding empirical priors from the previous state (*forward* message 2), the empirical priors from the subsequent state (*backward* message 3), and the *likelihood* (message 4). These messages are created by forward and backward matrix multiplications, enabling us to express belief propagation in terms of the sufficient statistics of the underlying probability distributions; namely, their expectations (see Figure 1, lower left panel):

(3) $$\begin{array}{l} \text{ln}\;{\text{s}}_{\pi ,\tau }=\text{ln}\;{\text{B}}_{\pi ,\tau -1}{\text{s}}_{\pi ,\tau -1}+\text{ln}\;{\text{B}}_{\pi ,\tau }\cdot {\text{s}}_{\pi ,\tau +1}+\text{ln}\;\text{A}\cdot o_{\tau }\Rightarrow \\ \;{\text{s}}_{\pi ,\tau }=\sigma \left(\text{ln}\;{\text{B}}_{\pi ,\tau -1}{\text{s}}_{\pi ,\tau -1}+\text{ln}\;{\text{B}}_{\pi ,\tau }\cdot {\text{s}}_{\pi ,\tau +1}+\text{ln}\;\text{A}\cdot o_{\tau }\right). \end{array}$$

The solution to this equality encodes posterior beliefs about hidden states. Here, *σ*(*·*) denotes the softmax operator, and backward matrix multiplication is denoted by the dot product **A***·* **s** = **A**^*T*^ **s**, where boldface matrices denote conditional (proper) probabilities such that

(4) $${\text{A}}_{ij}=\frac{A_{ij}}{\Sigma_{k}A_{kj}},\;{\text{A}}_{ij}^{T}=\frac{A_{ji}}{\Sigma_{k}A_{jk}},\;P(o_{\tau },s_{\tau })=Cat(A).$$

The same convention is used for the probability transitions matrices. The admixture of forward and backward messages in Eq. (2) renders this belief propagation akin to a Bayesian smoother or the forward-backward algorithm for hidden Markov models. However, unlike conventional schemes, the belief propagation here operates before seeing all the outcomes. In other words, expectations about hidden states are associated with successive time points during the enaction of a policy, equipping the model with a short-term memory of the past, and future. This means that a partially observed sequence of outcomes can inform expectations about the future, which are necessary to evaluate the expected free energy of a policy.

**Figure 2:**
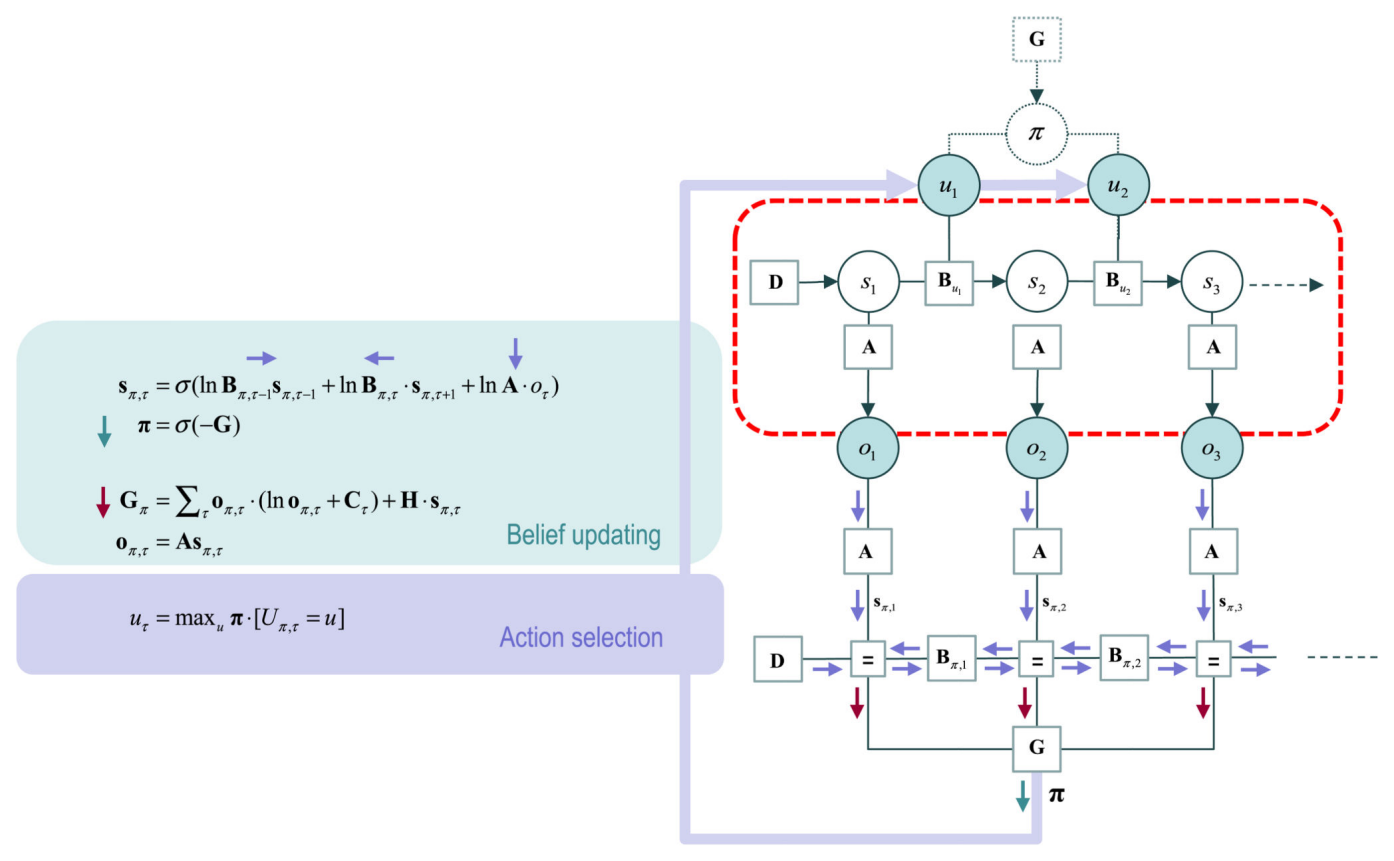
The generative process and model. This figure reproduces the Bayesian network and Forney factor graph of Figure 1. However, here the Bayesian network describes the process generating data, as opposed to the generative model of data. This means that we can link the two graphs to show how the policy half-edge of Figure 1 now couples back to the generative process (by generating an action that determines state transitions). The selected action corresponds to the most probable action under posterior beliefs about action sequences or policies. Here, the message labels have been replaced with little arrows to emphasize the circular causality implicit in active inference: The real world (red box) generates a sequence of outcomes that induce message passing and belief propagation to inform (approximate) posterior beliefs about policies (that also depend upon prior preferences and epistemic value). These policies then determine action, which generate new outcomes as time progresses, thereby closing the action perception cycle.

Figure 2 illustrates the recurrent nature of the message passing that mediates this predictive (and postdictive) inference using little arrows. One can see clearly that the first outcome can influence expectations about the final hidden state, and expectations about the final hidden state reach back and influence expectations about the initial state. This will become an important aspect of the deep temporal models considered later. In the present context, it means that we are dealing with loopy (cyclic) belief propagation because of the recurrent message passing. This renders the scheme approximate, as opposed to implementing exact Bayesian inference. It can be shown that the stationary point of iterative belief propagation in cyclic or loopy graphs minimizes (a marginal) free energy (Yedidia, Freeman, & Weiss, 2005). This highlights the close connection between variational message passing (Beal, 2003; MacKay, 2003), loopy belief propagation, and expectation propagation (Minka, 2001). The approximate nature of inference here rests on the fact that we are effectively optimizing marginal distributions over successive hidden states and are therefore approximating the real posterior with (see Figure 1)

(5) $$P(s_{1},\ldots \;,s_{T}|o_{1},\ldots \;,\pi )=\prod_{\tau }P(s_{\tau }|s_{\tau -1},\pi )\approx Q(s_{1},\ldots \;,s_{T}|\pi )=\prod_{\tau }Q(s_{\tau }|\pi ).$$

The corresponding *variational* free energy for this variational approximation is provided in the Supplementary Information, Appendix 2 (Friston, Parr, et al., 2017), and is formally related to the *marginal* free energy minimized by belief propagation or the sum-product algorithm described here (Friston, FitzGerald, Rigoli, Schwartenbeck, & Pezzulo, 2017; Yedidia et al., 2005).

Note that the Forney factor graph in Figure 1 posits separate messages for hidden states over time—and under each policy. This is consistent with what we know of neuronal representations; for example, distinct (place coded) representations of the past and future are implicit in the delay period activity shown in prefrontal cortical units during delayed matching to sample (Kojima & Goldman-Rakic, 1982); see Friston, FitzGerald, et al. (2017) for a discussion. Furthermore, separable (place coded) representations of policies are ubiquitous in the brain; for example, salience maps (Bender, 1981; Zelinsky & Bisley, 2015) or the encoding of (overt or covert) saccadic eye movements in the superior colliculus (Müller, Philiastides, & Newsome, 2005; Shen, Valero, Day, & Paré, 2011).

### The Active Bit of Active Inference

Figure 2 combines Bayesian and Forney factor graphs to distinguish between the process generating outcomes and the concomitant inference that the outcomes induce. Crucially, the Bayesian network describing the *generative process* is not the Bayesian network describing the *generative model*, upon which the factor graph is based. In other words, in active inference the generative model and process are distinct. This is because the actual causes of data depend upon action and action depends upon inference (about policies). In other words, the end point of inference is a belief about policies that specify actions—and actions affect the transitions among the true states generating data. In short, the inference scheme effectively chooses the data it uses for inference. This means that the hidden policies do not actually exist; they are fictive constructs that are realized through action. This is an important part of active inference and shows how policies are coupled to the real world through action to complete the perception and action cycle. It also highlights the circular causality mediated by message passing: Messages flow from outcome nodes to a factor corresponding to expected free energy that determines the probability distribution over policies, thereby producing purposeful (epistemic and goal-directed) behavior. In summary, there are several useful insights into the computational architecture afforded by graphical representations of belief propagation. However, what does this tell us about the brain?

### Belief Propagation and Neuronal Dynamics

**Figure 3:**
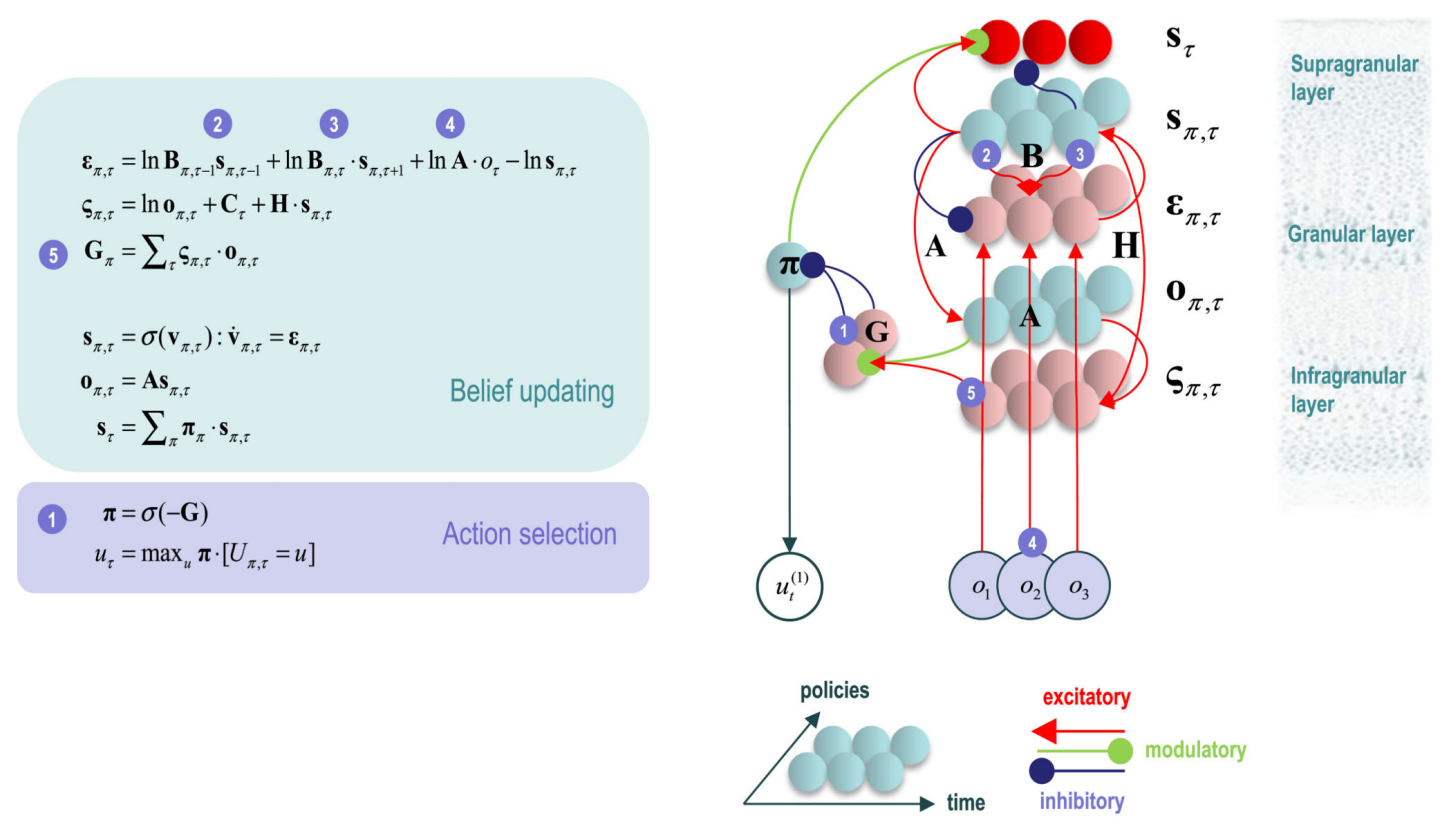
Belief propagation and intrinsic connectivity. **Right panel:** The schematic on the right represents the message passing implicit in the differential (update) equations on the left. The expectations have been associated with neuronal populations (colored balls) that are arranged to reproduce known intrinsic (within cortical area) connections. Red connections are excitatory, blue connections are inhibitory, and green connections are modulatory (i.e., involve a multiplication or weighting). Here, edges correspond to intrinsic connections that mediate the message passing on the left. Cyan units correspond to expectations about hidden states and (future) outcomes under each policy, while red states indicate their Bayesian model averages. Pink units correspond to (state and outcome) prediction errors that are averaged to evaluate expected free energy and subsequent policy expectations (in the lower part of the network). This (neural) network formulation of belief updating means that connection strengths correspond to the parameters of the generative model in Figure 1. Only exemplar connections are shown to avoid visual clutter. Furthermore, we have just shown neuronal populations encoding of hidden states under two policies over three time points (i.e., two transitions). **Left panel:** The differential equations on the left share the same stationary solution as the belief updates in previous figures and can therefore be interpreted as a gradient descent on (marginal) free energy. The equations have been expressed in terms of prediction errors that come in two flavors. The first, *state* prediction error, scores the difference between the (logarithms of) expected states under each policy and time point—and the corresponding predictions based upon outcomes and the (preceding and subsequent) hidden states. These represent empirical prior and likelihood terms respectively; namely, *messages* 2, 3, and 4. The prediction error drives depolarization in state units, where the expectation per se is obtained via a softmax operator. The second, *outcome* prediction error, reports the difference between the (logarithms of) expected outcomes and those predicted under prior beliefs. This prediction error is weighted by the expected outcomes to evaluate the expected free energy **G**, via *message* 5. These policy-specific free energies are combined to give the policy expectations via a softmax function, via *message* 1.

A robust and dynamic belief (or expectation) propagation scheme can be constructed easily by setting up ordinary differential equations whose solution satisfies Equation 3, whereby, substituting ***ν**_π,τ_* = ln *s_π,τ_* and introducing an auxiliary variable (state prediction error), one obtains the following update scheme (see also Figure 3):

(6) $$\begin{array}{l} \varepsilon_{\pi ,\tau }=\text{ln}\;{\text{B}}_{\pi ,\tau -1}{\text{s}}_{\pi ,\tau -1}+\text{ln}\;{\text{B}}_{\pi ,\tau }.{\text{s}}_{\pi ,\tau +1}+\text{ln}\;\text{A}\cdot o_{\tau }-\text{ln}\;{\text{s}}_{\pi ,\tau }\\ {\dot{\upsilon }}_{\pi ,\tau }=\varepsilon_{\pi ,\tau }\\ {\text{s}}_{\pi ,\tau }=\sigma ({\text{v}}_{\pi ,\tau }). \end{array}$$

These differential equations correspond to a gradient descent on (marginal) variational free energy as described in Friston, FitzGerald, et al. (2017):

(7) $${\dot{\text{v}}}_{\pi ,\tau }=\varepsilon_{\pi ,\tau }=-\frac{\partial {\text{F}}_{\pi }}{\partial {\text{s}}_{\pi ,\tau }}.$$

Crucially, they also furnish a process theory for neuronal dynamics, in which the sigmoid function can be thought of as playing the role of a sigmoid (voltage – firing rate) activation function. This means log expectations about hidden states can be associated with depolarization of neurons or neuronal populations encoding expectations. The key point here is that belief propagation entails simple operations that map onto the operators commonly employed in neural networks; namely, the convergence of (mixtures of) presynaptic input, in terms of (nonnegative) firing, to mediate a postsynaptic depolarization—that then produces a firing rate response through a nonlinear activation function. This is the (convolution) form of neural mass models of population activity used to model electrophysiological responses (for example, Jansen & Rit, 1995). This formulation also has some construct validity in relation to theoretical proposals and empirical work on evidence accumulation (de Lafuente, Jazayeri, & Shadlen, 2015; Kira, Yang, & Shadlen, 2015) and the neuronal encoding of probabilities (Deneve, 2008). Interestingly, it also casts prediction error as a free energy gradient, which is effectively destroyed as the gradient descent reaches its attracting (stationary) point; see Tschacher and Haken (2007) for a synergetic perspective.

### Canonical Microcircuits for Belief Propagation

The neural network in Figure 3 tries to align the message passing in the Forney factor graph with quantitative studies of intrinsic connections among cortical layers (Thomson & Bannister, 2003). This (speculative) assignment allows one to talk about the functional anatomy of intrinsic connectivity in terms of belief propagation. In this example, state prediction error units (pink) have been assigned to granular layers (e.g., spiny stellate populations) that are in receipt of ascending sensory information (the *likelihood* message). These project to units in supragranular layers that represent (policy specific) expected states (cyan), which are averaged to form Bayesian model averages—associated with superficial pyramidal cells (red). The expected states then send forward (intrinsic) connections to units encoding expected outcomes in infragranular layers, which in turn excite outcome prediction errors (necessary to evaluate expected free energy; see Appendix 2: Friston, Parr, et al., 2017). This nicely captures the forward (generally excitatory) intrinsic connectivity from granular, to supragranular, to infragranular populations that characterize the canonical cortical microcircuit (Bastos et al., 2012; Douglas & Martin, 1991; Haeusler & Maass, 2007; Heinzle, Hepp, & Martin, 2007; Shipp, 2016). Note also that reciprocal (backward) intrinsic connections from the expected states to state prediction errors are inhibitory, suggesting that both excitatory and inhibitory interneurons in the supragranular layer encode (policy specific) expected states. Computationally, Equation 6 suggests this (interlaminar) connection is inhibitory because the last contribution (from expected states) to the prediction error is negative. Neurobiologically, this may correspond to a backward intrinsic pathway that is dominated by projections from inhibitory interneurons (Haeusler & Maass, 2007).

The particular formulation in Equation 6 distinguishes between the slower dynamics of populations encoding expectations of hidden states and the instantaneous responses of populations encoding prediction errors. This formulation leads to interesting hypotheses about the characteristic membrane time constants of spiny stellate cells encoding prediction errors, relative to pyramidal cells encoding expectations (e.g., Ballester-Rosado et al., 2010). Crucially, because prediction errors are a function of expectations and the *rates of change* of expectations are functions of prediction errors, one would expect to see the same sort of spectral differences in laminar-specific oscillations that have been previously discussed in the context of predictive coding (Bastos et al., 2012, 2015; Bosman et al., 2012).

This and subsequent neural networks should not be taken too seriously; they are offered as a starting point for refinement and deconstruction, based upon anatomy and neurophysiology. See Shipp (2016) for a nice example of this endeavor. The neural network above inherits a lot of its motivation from similar (more detailed) arguments about the laminar specificity of neuronal message passing in canonical microcircuits implied by predictive coding. Fuller accounts of the anatomical and neurophysiological evidence—upon which these arguments are based—can be found in Adam, Shipp, and Friston (2013), Bastos et al. (2012), Friston (2008), Mumford (1992), Shipp (2005, 2016), and Shipp, Adam, and Friston (2013). See Whittington and Bogacz (2017) for treatment that focuses on the role of intralaminar connectivity in learning and encoding uncertainty. One interesting component that the belief propagation scheme brings to the table (that is not in predictive coding; see Figures 7 and 10 below) is the encoding of outcome prediction errors in deep layers that send messages to (subcortical) nodes encoding expected free energy. This message passing could be mediated by corticostriatal projections, from layer 5 (and deep layer 3) pyramidal neurons, which are distributed in a patchy manner (Haber, 2016). We now move from local (intrinsic) message passing to consider the basic form of hierarchical message passing, of the sort that might be seen in cortical hierarchies.

### Deep Temporal Models

**Figure 4:**
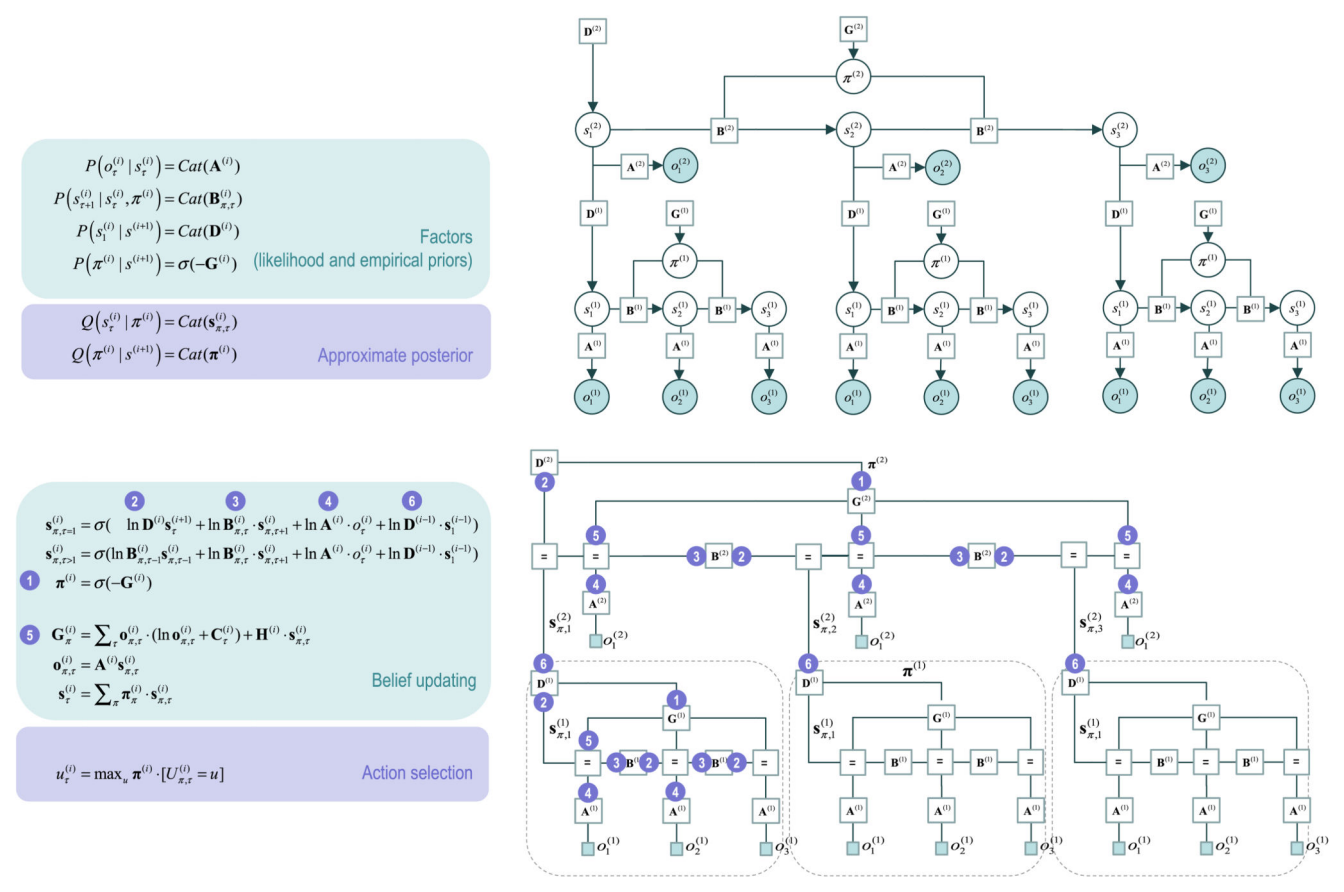
Deep generative models. This figure provides the Bayesian network and associated Forney factor graph for deep (temporal) generative models, described in terms of probability factors and belief updates on the left. The graphs adopt the same format as Figure 1; however, here the previous model has been extended hierarchically, where (bracketed) superscripts index the hierarchical level. The key aspect of this model is its hierarchical structure that represents sequences of hidden states over time or epochs. In this model, hidden states at higher levels generate the initial states for lower levels—that then unfold to generate a sequence of outcomes; cf. associative chaining (Page & Norris, 1998). Crucially, lower levels cycle over a sequence for each transition of the level above. This is indicated by the subgraphs enclosed in dashed boxes, which are “reused” as higher levels unfold. It is this scheduling that endows the model with deep temporal structure. In relation to the (shallow) models above, the probability distribution over initial states is now conditioned over the state (at the current time) of the level above. Practically, this means that **D** now becomes a matrix, as opposed to a vector. The messages passed from the corresponding factor node rest on Bayesian model averages that require the expected policies (*message* 1) and expected states under each policy. The resulting averages are then used to compose descending (*message* 2) and ascending (*message* 6) messages that mediate the exchange of empirical priors and posteriors between levels respectively.

The generative model in Figure 1 considers only a single timescale or temporal horizon specified by the depth of policies entertained. Clearly, the brain models the temporal succession of worldly states at multiple timescales, calling for hierarchical or deep models. An example is provided in Figure 4 that effectively composes a deep model by diverting some (or all) of the outputs of one model to specify the initial states of another (subordinate) model, with exactly the same form. The key aspect of this generative model is that state transitions proceed at different rates at different levels of the hierarchy. In other words, the transition from one hidden state to the next entails a sequence of transitions at the level below. This is a necessary consequence of conditioning the initial state at any level on the hidden states in the level above. Heuristically, this hierarchical model generates outcomes over nested timescales, like the second hand of a clock that completes a cycle for every tick of the minute hand that precesses more quickly than the hour hand. It is this particular construction that lends the generative model a deep temporal architecture. In other words, hidden states at higher levels contextualize transitions or trajectories of hidden states at lower levels to generate a deep narrative.

In terms of message passing, the equivalent Forney factor graph (Figure 4: lower right) shows that the message passing within each level of the model is conserved. The only difference is that messages are sent in both directions along the edge connecting the factor (**D**) representing the joint distribution over the initial state conditioned upon the state of the level above. These messages correspond to *ascending* and *descending* messages, respectively. The ascending message 6 effectively supplements the observations of the level above using the (Bayesian model) average of the states at the level below. Conversely, the descending message 2 plays a role of an empirical prior that induces expectations about the initial state of the level below, thereby contextualizing fast subordinate sequences within slower supraordinate narratives.

Crucially, the requisite Bayesian model averages depend on expected policies via message 1. In other words, the expected states that constitute descending priors are a weighted mixture of policy-specific expectations that, incidentally, mediate a Bayes optimal optimism bias (Friston et al., 2013; Sharot, Guitart-Masip, Korn, Chowdhury, & Dolan, 2012). This Bayesian model averaging lends expected policies a role above and beyond action selection. This can be seen from the Forney factor graph representation, which shows that messages are passed from the expected free energy node **G** to the initial state factor **D**. In short, policy expectations now exert a powerful influence over how successive hierarchical levels talk to each other. We will pursue this later from an anatomical perspective in terms of extrinsic connectivity and cortico–basal ganglia–thalamic loops. Before considering the implications for hierarchical architectures in the brain, we turn to the equivalent message passing for continuous variables, which transpires to be predictive coding (Rao & Ballard, 1999; Srinivasan, Laughlin, & Dubs, 1982).

## Models for Continuous States

This section rehearses the treatment of the previous section using models of continuous states. We adopt a slightly unusual formulation of continuous states that both generalizes established Bayesian filtering schemes and, happily, has a similar form to generative models for discrete states. This generalized form rests upon describing trajectories in *generalized coordinates of motion*. These are common in physics (e.g., position and momentum). Here, we consider motion to arbitrarily high order (i.e., location, velocity, acceleration, jerk). A key thing to bear in mind, when dealing with generalized motion, is that the mean of the generalized motion is the motion of the mean when, and only when, free energy is minimized. This corresponds to Hamilton’s principle of least action.

**Figure 5:**
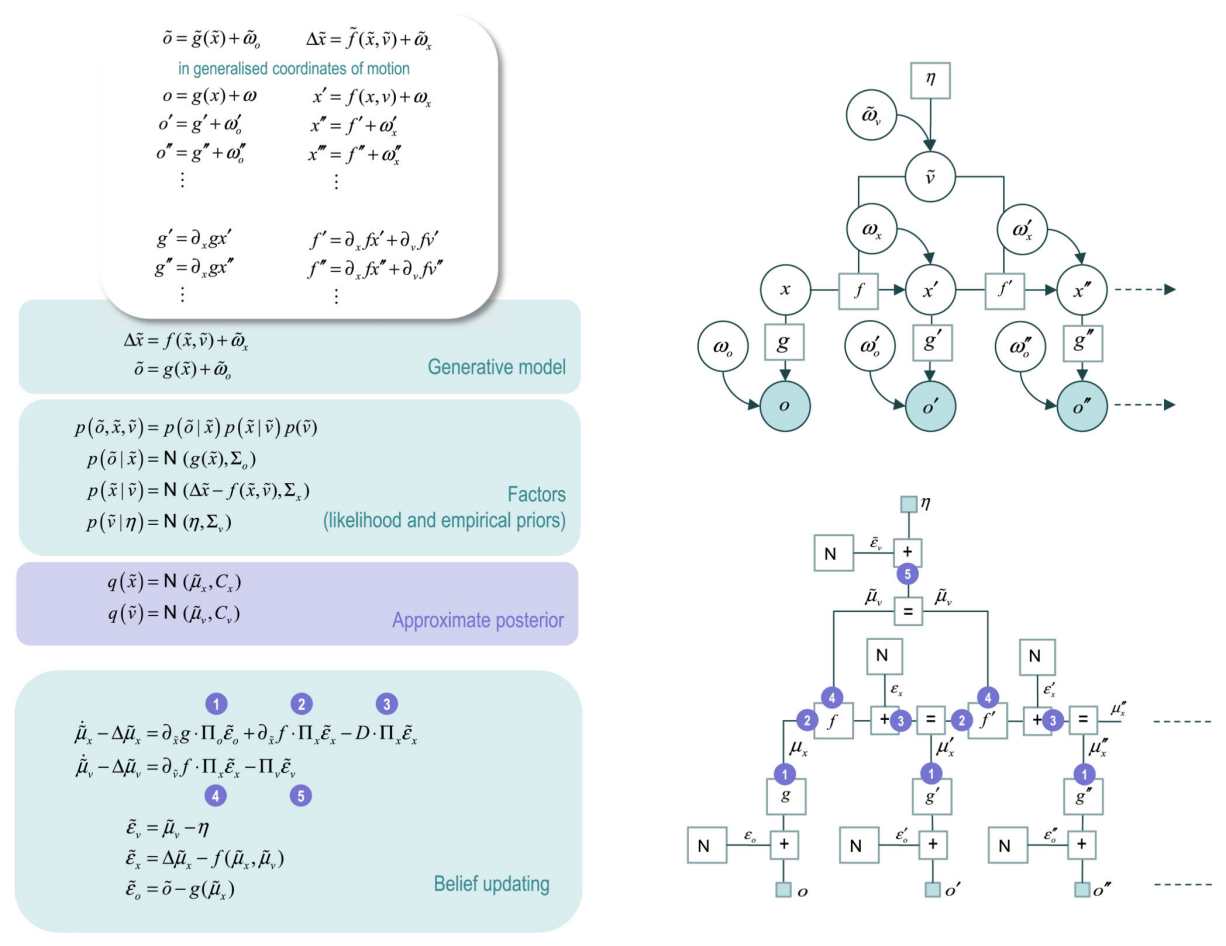
A generative model for continuous states. This figure describes a generative model for a continuous state space model in terms of generalized motion, using the same format as Figure 1. Here, outcomes are generated (with a static nonlinear mapping *g*) from the current state, with some random fluctuations; similarly for higher orders of motion. The motion of hidden states is a function (equation of motion or flow *f*) of the current state, plus random fluctuations. Higher-order motion is derived in a similar way using generalized equations of motion (in the upper left inset). The corresponding Bayesian network (on the upper right) shows that discrete probability transition matrices are replaced with generalized equations of motion, while the discrete likelihood mapping becomes the static nonlinearity. The corresponding Forney factor graph is shown on the lower right.

Figure 5 shows a generative model for a short sequence or trajectory described in terms of generalized motion. The upper panel (on the left) shows that an outcome is generated (with a static nonlinear mapping *g*) from the current state, with some random fluctuations; similarly for higher orders of motion. The motion of hidden states is a function (equation of motion or flow *f*) of the current state, plus random fluctuations. Higher-order motion is derived in a similar way using generalized equations of motion (in the upper left inset). The Bayesian network (on the upper right) has been written in a way that highlights its formal similarity with the equivalent network for discrete states (Figure 1). This entails lumping together the generalized hidden causes that are generated from a prior expectation. In this form, one can see that the probability transition matrices are replaced with generalized equations of motion, while the likelihood mapping becomes the static nonlinearity. The corresponding Forney factor graph is shown on the lower right, where we have introduced some special (local function) nodes corresponding to factors generating random fluctuations and their addition to predictions of observed trajectories and the flow of hidden states.

Following the derivation of the belief propagation for discrete states, we can pursue a similar construction for continuous states to derive a generalized (Bayesian or variational) filter. For example, using the notation *x̃* = (*x*, *x′*, *x″*, …) = (*x*^[0]^, *x*^[1]^, *x*^[2]^, …) for generalized states,

(8) $$\begin{array}{c} \;q\left(x^{\left[i\right]}\right)\propto \mu_{g^{\left[i\right]}\to x^{\left[i\right]}}\cdot \mu_{f^{\left[i\right]}\to x^{\left[i\right]}}\cdot \mu_{f^{\left[i-1\right]}\to x^{\left[i\right]}}\Rightarrow \\ \;\text{ln}\;q\left(x^{\left[i\right]}\right)=\underset{\text{likelihood}\;(1)}{\underset{︸}{\text{ln}\;\mu_{g^{\left[i\right]}\to x^{\left[i\right]}}}}+\underset{\text{backward}\;(2)}{\underset{︸}{\text{ln}\;\mu_{f^{\left[i\right]}\to x^{\left[i\right]}}}}+\underset{\text{forward}\;(3)}{\underset{︸}{\text{ln}\;\mu_{f^{\left[i-1\right]}\to x^{\left[i\right]}}}}\\ \;=-\frac{1}{2}\left(\varepsilon_{o}^{\left[i\right]}.\Pi_{o}^{\left[i\right]}\varepsilon_{o}^{\left[i\right]}+\varepsilon_{x}^{\left[i\right]}\cdot \Pi_{x}^{\left[i\right]}\varepsilon_{x}^{\left[i\right]}+\varepsilon_{x}^{\left[i-1\right]}\cdot \Pi_{x}^{\left[i-1\right]}\varepsilon_{x}^{\left[i-1\right]}\right)\\ \;\varepsilon_{x}^{\left[i\right]}\;=x^{\left[i+1\right]}-f^{\left[i\right]}\left(x^{\left[i\right]},v^{\left[i\right]}\right)\\ \varepsilon_{o}^{\left[i\right]}\;=o_{o}^{\left[i\right]}-g^{\left[i\right]}(x^{\left[i\right]}). \end{array}$$

At the posterior expectation, the derivative of this density with respect to the unknown states must be zero; therefore,

(9) $$\begin{array}{l} \partial_{x^{\left[i\right]}}\text{ln}\;q\left(\mu_{x}^{\left[i\right]}\right)=\partial_{x^{\left[i\right]}}g^{\left[i\right]}\cdot \Pi_{o}^{\left[i\right]}\varepsilon_{o}^{\left[i\right]}+\partial_{x^{\left[i\right]}}f^{\left[i\right]}\cdot \Pi_{x}^{\left[i\right]}\varepsilon_{x}^{\left[i\right]}+\Pi_{x}^{\left[i-1\right]}\varepsilon_{x}^{\left[i-1\right]}\\ \;\Leftrightarrow \\ \;\partial_{\tilde{x}}\text{ln}\;q\left({\tilde{\mu }}_{x}\right)=\partial_{\tilde{x}}\tilde{g}\cdot \Pi_{o}{\tilde{\varepsilon }}_{o}+\partial_{\tilde{x}}\tilde{f}\cdot \Pi_{x}{\tilde{\varepsilon }}_{x}-\Delta \cdot \Pi_{x}{\tilde{\varepsilon }}_{x}=0\\ \;{\tilde{\varepsilon }}_{x}=\Delta {\tilde{\mu }}_{x}-\tilde{f}\left({\tilde{\mu }}_{x},{\tilde{\mu }}_{v}\right)\\ \;{\tilde{\varepsilon }}_{o}=\tilde{o}-\tilde{g}({\tilde{\mu }}_{x}). \end{array}$$

Here, the (block matrix) operator Δ returns higher-order motion Δ*x̃* = (*x′*, *x″*, *x‴*, …), while its transpose returns lower order motion Δ · *x̃* = (0, *x′*, *x″*, …). As before, we can now construct a differential equation whose solution satisfies the above equality:

(10) $${\dot{\tilde{\mu }}}_{x}-\Delta {\tilde{\mu }}_{x}=\partial_{\tilde{x}}\tilde{g}\cdot \Pi_{o}{\tilde{\varepsilon }}_{o}+\partial_{\tilde{x}}\tilde{f}\cdot \Pi_{x}{\tilde{\varepsilon }}_{x}-\Delta \cdot \Pi_{x}{\tilde{\varepsilon }}_{x}.$$

The solution of this equation ensures that when Equation 9 is satisfied; the motion of the mean is the mean of the motion ${\dot{\tilde{\mu }}}_{x}=\Delta {\tilde{\mu }}_{x}.$ Crucially, this equation is a generalized gradient descent on variational free energy (Friston et al., 2010; Friston, 2008):

(11) $${\dot{\tilde{\mu }}}_{x}=\Delta {\tilde{\mu }}_{x}-\partial_{\tilde{x}}F.$$

In this instance, belief propagation and variational message passing (i.e., variational filtering) are formally identical, because the messages in belief propagation are the same as those required from the Markov blanket of each random variable in the corresponding Bayesian network (Beal, 2003; Dauwels, 2007; Friston, 2008).

**Figure 6:**
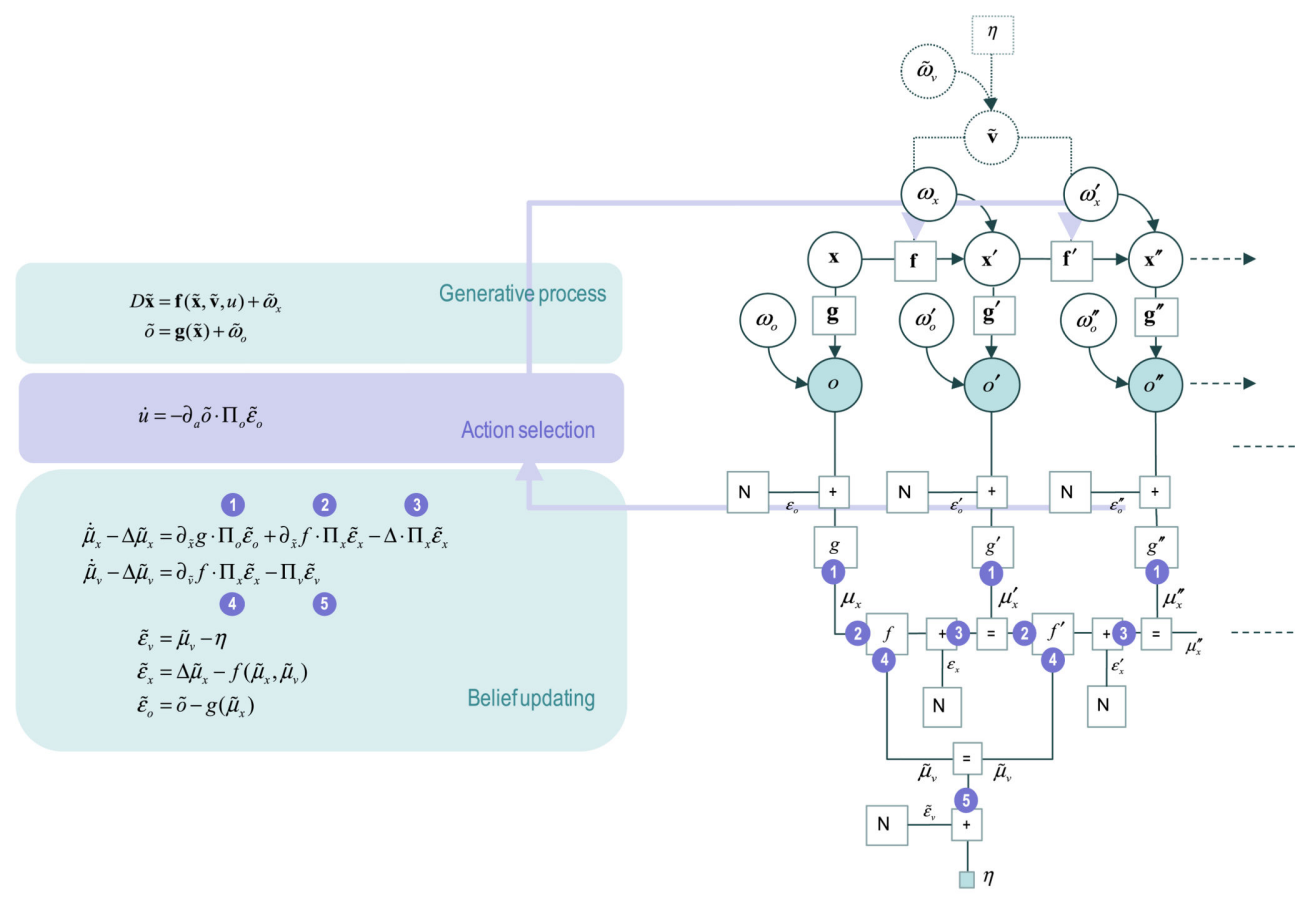
Active inference with continuous states (and time). This figure uses the same format as Figure 2 to illustrate how action couples to the generative process. As before, action supplements or replaces hidden causes in the generative model—to complete the generative process. In this instance, action minimizes variational free energy directly, as opposed to minimizing expected free energy via inference over policies. For simplicity, we have removed any dependency of the observation on causes. This dependency is reinstated in subsequent figures.

The ensuing generalized variational or Bayesian filtering scheme has several interesting special cases, including the (extended) Kalman (Bucy) filter, which falls out when we only consider generalized motion to first order. See Friston et al. (2010) for details. When expressed in terms of prediction errors, this generalized variational filtering corresponds to predictive coding (Rao & Ballard, 1999; Srinivasan et al., 1982) that has become an accepted metaphor for evidence accumulation in the brain. In terms of active inference, the minimization of free energy with respect to action or control states only has to consider the prediction errors on outcomes (because these are the only things that can be changed by action). This leads to the active inference scheme in Figure 6.

As with the discrete models, action couples back to the generative process through affecting state transitions or flow. Note, as above, the generative process can be formally distinct from the generative model. This means the real equations of motion (denoted by boldface functions **f**) become functions of action. In this context, one can see how the (fictive) hidden causes in the generative model are replaced by (or supplemented with) action; that is a product of inference. The joint minimization of free energy—or maximization of model evidence—by action and perception rests upon the implicit closure of conditional dependencies between the process (world) and model (brain). See K. Friston (2011) and K. Friston, Mattout, and Kilner (2011) for more details.

### Canonical Microcircuits for Predictive Coding

**Figure 7:**
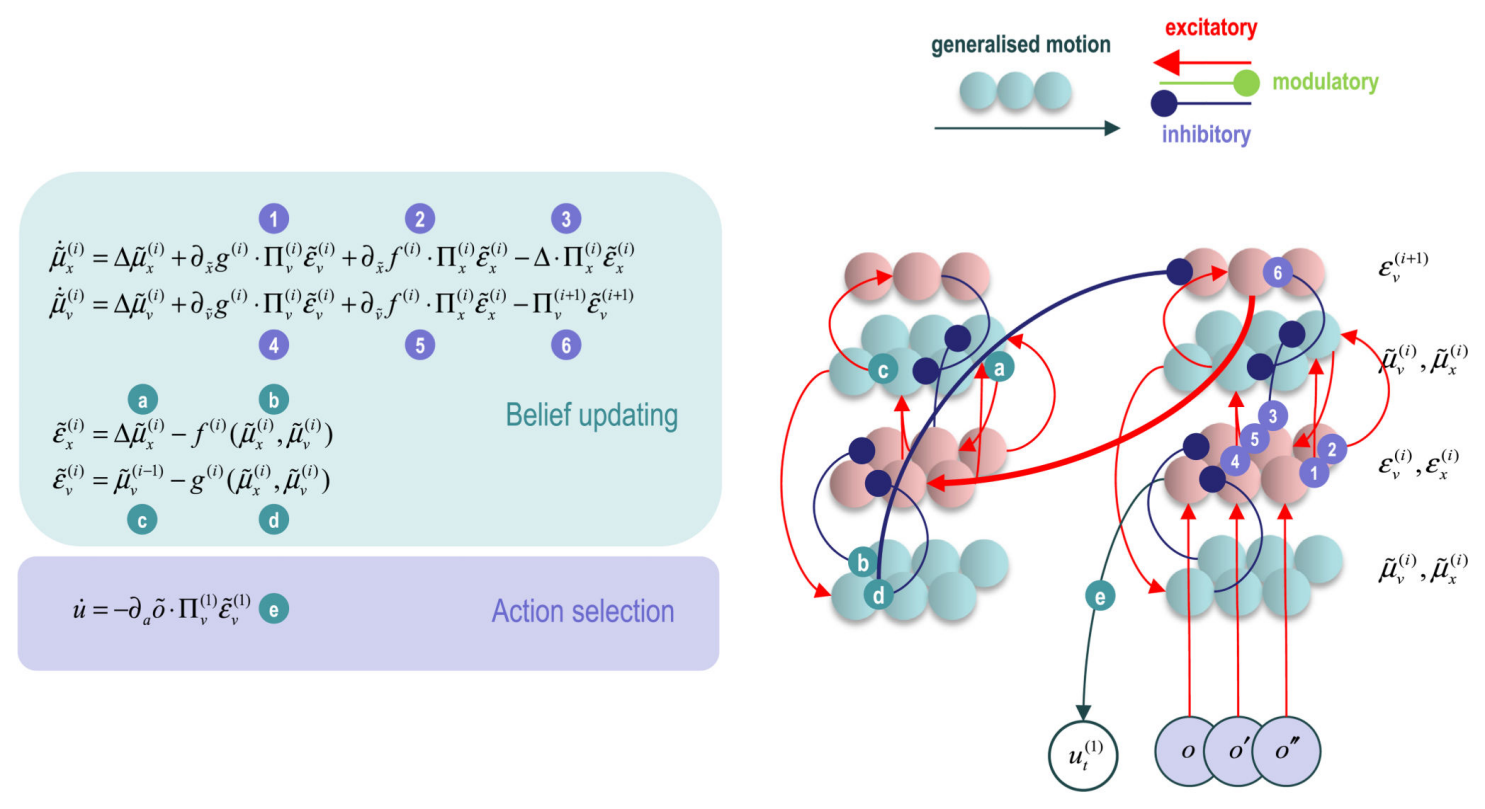
Canonical microcircuits for predictive coding. This proposal is based upon the anatomy of intrinsic and extrinsic connections described in Bastos et al. (2012). This figure provides the update dynamics for a hierarchical generalization of the generative model in Figure 6, where the outputs of a higher level now become the hidden causes of the level below. In this hierarchical setting, the prediction errors include prediction errors on both hidden causes and states. As with the model for discrete states, the prediction errors have been assigned to granular layers that receive sensory afferents and ascending prediction errors from lower levels in the hierarchy.

Figure 7 depicts a neural network that might implement the message passing in Figure 6. This proposal is based upon the anatomy of intrinsic and extrinsic connections described in Bastos et al. (2012). This figure provides the update dynamics for a hierarchical generalization of the generative model in Figure 6, where the outputs of a higher level now become the hidden causes of the level below. In this hierarchical setting, the prediction errors include prediction errors on both hidden causes and states. As with the model for discrete states, the prediction errors have been assigned to granular layers that receive sensory afferents and ascending prediction errors from lower levels in the hierarchy. Given that the message passing requires prediction errors on hidden causes to be passed to higher levels, one can assume they are encoded by superficial pyramidal cells; that is, the source of ascending extrinsic connections (Bastos et al., 2012; Felleman & Van Essen, 1991; Markov et al., 2013). Similarly, the sources of descending extrinsic connections are largely restricted to deep pyramidal cells that can be associated with expected hidden causes. The ensuing neural network is again remarkably consistent with the known microcircuitry of extrinsic and intrinsic connections, with a series of forward (intrinsic connections from granular, to supragranular, to infragranular layers) and reciprocal inhibitory connections (Thomson & Bannister, 2003). A general prediction of this computational architecture is that no pyramidal cell (early in cortical hierarchies) can be the source of both forward and backward connections. This follows from the segregation of neuronal populations encoding expectations and errors respectively, where error units send ascending projections to the granular layers of a higher area, while pyramidal cells encoding expectations project back to error units in lower areas. This putative law of extrinsic connectivity has some empirical support as reviewed in Shipp (2016).

The main difference between the microcircuits for discrete and continuous states is that the superficial pyramidal cells encode state *expectations* and *prediction errors* respectively. This is because in discrete belief propagation ascending and descending messages convey posteriors (i.e., expected states computed by the lower level) and empirical priors (i.e., the states predicted by the higher level) respectively; whereas, in predictive coding, they convey prediction errors and predictions respectively. Furthermore, the encoding of expected outcomes is not necessary for predictive coding. This is because we have replaced policies with hidden causes. One might ask how predictive coding can support epistemic and purposeful behavior in the absence of policies that underwrite behavior. The answer pursued below is that the hidden causes (of continuous state models) are provided by the policy-rich predictions of (discrete state) models. In the next section, these enriched predictions are considered in terms of message passing and salience.

## Mixed Models

**Figure 8:**
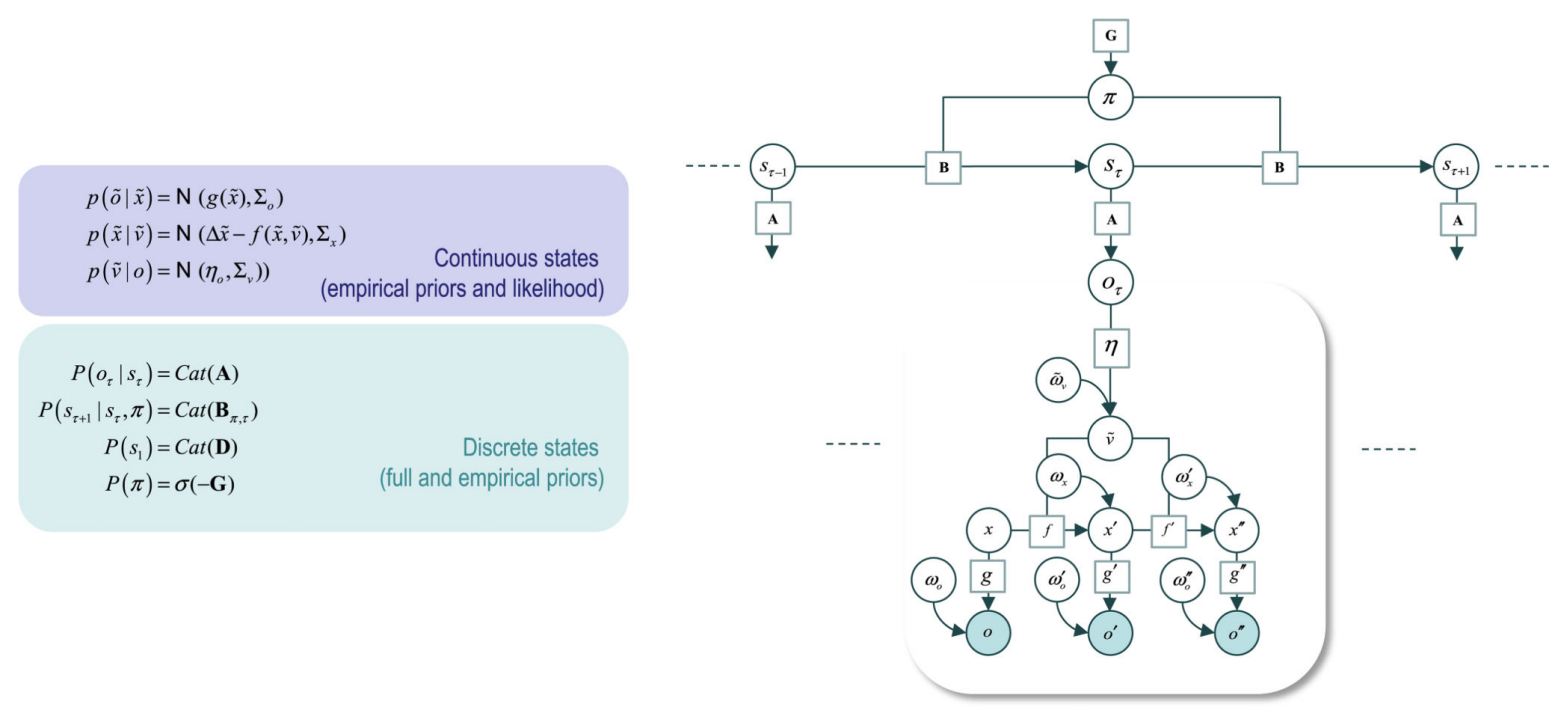
Linking discrete and continuous state models. This figure uses the same format as previous figures but combines the *discrete* Bayesian network in Figure 1 with the *continuous* Bayesian network from Figure 5. Here, the outcomes of the discrete model are now used to select a particular (noisy generalized) hidden cause that determines the (noisy generalized) motion or flow of hidden states generating (noisy generalized) observations. These generalized observations described a trajectory in continuous time.

This section considers the integration of discrete and continuous models, and what this implies for message passing in neuronal circuits. In brief, we will see that discrete outcomes select a specific model of continuous trajectories or dynamics—as specified by a prior over their hidden causes. Effectively, this generative model generates (discrete) sequences of short (continuous) trajectories defined in terms of their generalized motion. See Figure 8. Because state transitions occur discretely, the hidden causes generating dynamics switch periodically. This sort of model, if used by the brain, suggests the sensorium is constructed from discrete sequences of continuous dynamics (see also Linderman et al., 2016, for a recent machine learning perspective on this scenario). An obvious example here would be a sequence of saccadic eye movements, each providing a sample of the visual world and yet each constituted by carefully crafted oculomotor kinetics. In fact, this is an example that we will use below, where the discrete outcomes or priors on hidden causes specify a fixed-point attractor for proprioceptive (oculomotor) inference. In essence, this enables the discrete model to prescribe salient points of attraction for visual sampling.

**Figure 9:**
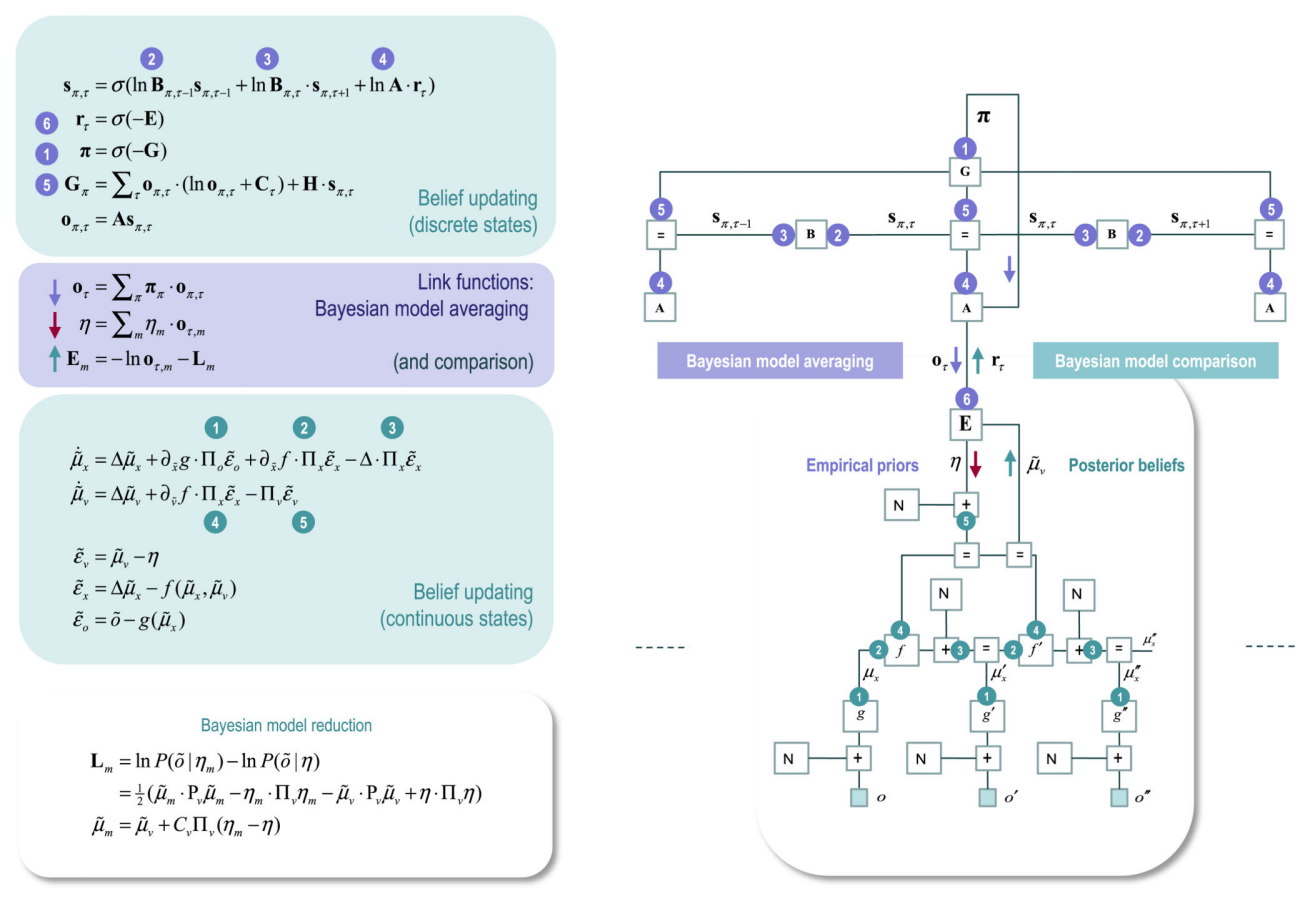
Mixed message passing. This figure combines the Forney factor graphs from Figures 1 and 5 to create a message passing scheme that integrates discrete and continuous models. The key aspect of this integration is the role of a link node (**E**) associated with the distribution over discrete outcomes—that can also be considered as (outcome) models from the perspective of the (subordinate) continuous level. The consequent message passing means that descending signals from the discrete to continuous levels comprise empirical prior beliefs about the dynamics below, while ascending messages constitute the posterior beliefs over the same set of outcome models. The corresponding messages are shown with little arrows that are reproduced alongside the link functions in the left panels. These linking updates entail two sorts of (Bayesian model) averages. First, expected outcomes under each policy are averaged over policies. Second, prior expectations about hidden causes are averaged over outcome models. These constitute the descending empirical priors. Conversely, ascending messages correspond to the posterior over outcomes models based upon a post hoc Bayesian model comparison. This treats each discrete hidden cause as a different model. The expressions in the lower left inset define the log likelihood of the continuous observations under each outcome model, relative to the log likelihood under their Bayesian model average.

In terms of belief propagation, Figure 9 shows that the descending messages comprise Bayesian model averages of predicted outcomes, while ascending messages from the lower, continuous, level of the hierarchical model are the posterior estimate of these outcomes, having sampled some continuous observations. In other words, the descending messages provide empirical priors over the dynamics of the lowest (continuous) level that returns the corresponding posterior distribution. This posterior distribution is interesting because it constitutes a Bayesian model comparison. This follows because we have treated each outcome as a particular model of dynamics, defined by plausible priors over hidden causes. Therefore, the posterior distribution over priors corresponds to the posterior probability for each model, given the continuous data at hand. From the point of view of the discrete model, each dynamic model corresponds to an outcome. From the point of view of the continuous level, each dynamic model corresponds to a particular prior on hidden causes.

The evaluation of the evidence for each alternative prior (i.e., outcome model) rests upon recent advances in post hoc model comparison. This means that ascending message can be computed directly (under the Laplace assumption) from the posterior over hidden causes and the prior (afforded by the descending message). In Figure 9, this is expressed as a softmax function of the free energy associated with each outcome model or prior. In practice, the dynamical system is actually integrated over a short period of time (about 200 ms in the examples below). This means the descending message corresponds to (literally) evidence accumulation over time:

(12) $$\begin{array}{l} \text{E}(t{)}_{m}=-\text{ln}\;{\text{o}}_{\tau ,m}-\int_{0}^{T}\text{L}{\left(t\right)}_{m}dt\\ \text{L}(t{)}_{m}=\text{ln}\;P(\tilde{o}(t)\eta_{m})-\text{ln}\;P(\tilde{o}(t)\eta ). \end{array}$$

This equation expresses the free energy **E** of competing outcome models as the prior surprise plus the log evidence for each outcome model (integrated over time). The log evidence is a relatively simple function of the posterior and prior (Gaussian) probability densities used to sample continuous observations and the prior that defines each outcome model. See K. Friston and Penny (2011) and Hoeting et al. (1999) for details. Note that if the posterior expectation coincides with the prior, its (relative) log evidence is zero (see the expression in Figure 9). In other words, the free energy for each outcome model *m* scores its “goodness” in relation to the Bayesian model average (over predicted discrete models). Note further that if duration of evidence accumulation shrinks to zero (*T* = 0), the ascending posterior reduces to the descending prior.

In summary, the link factor that enables one to combine continuous and discrete (hierarchical) models corresponds to a distribution over models of dynamics—and mediates the transformation of descending model averages into ascending model posteriors—using Bayesian model averaging and comparison respectively. Effectively, this equips the associated belief propagation scheme with the ability to categorize continuous data into discrete categories and, in the context of the deep temporal models considered here, chunk continuous sensory flows into discrete sequential representations. We will exploit this faculty in simulations of reading below. However, first we consider the implications of this hierarchical generative model for extrinsic (hierarchical) message passing in the brain.

### Extrinsic Connectivity in the Brain

**Figure 10:**
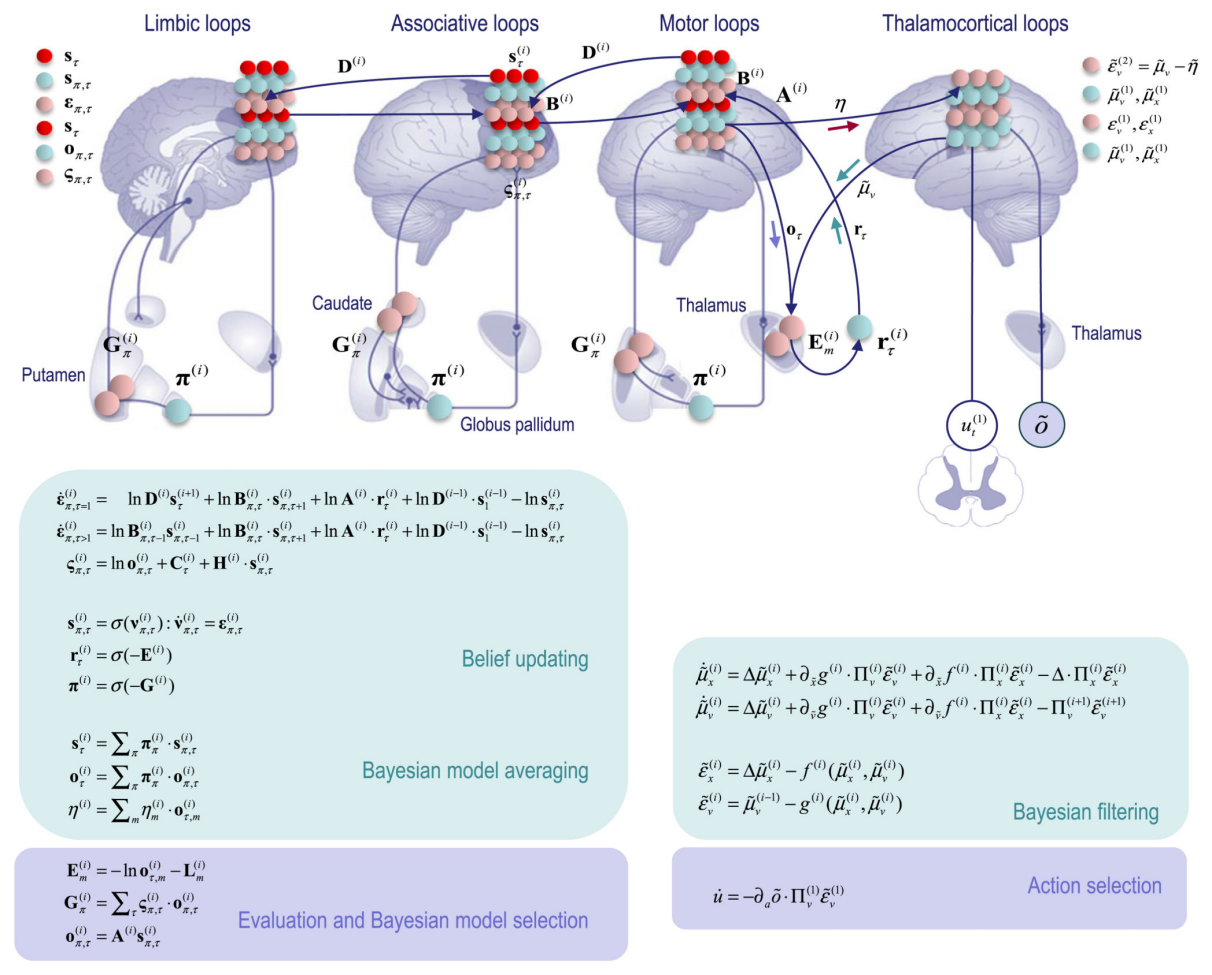
Deep architectures in the brain. This schematic illustrates a putative mapping of belief updating onto recurrent interactions within the cortico (basal ganglia) thalamic loops. This neural architecture is based upon the functional neuroanatomy described in Jahanshahi et al. (2015), which assigns motor updates to motor and premotor cortex projecting to the putamen, associative loops to prefrontal cortical projections to the caudate, and limbic loops to projections to the ventral striatum. The striatum (caudate and putamen) receive inputs from several cortical and subcortical areas. The internal segment of the globus pallidus constitutes the main output nucleus from the basal ganglia. The basal ganglia are connected to motor areas (motor cortex, supplementary motor cortex, premotor cortex, cingulate motor area, and frontal eye fields) and associative cortical areas. The basal ganglia nuclei have similar (topologically organized) motor, associative, and limbic territories; the posterior putamen is engaged in sensorimotor function, while the anterior putamen (or caudate) and the ventral striatum are involved in associative (cognitive) and limbic (motivation and emotion) functions (Jahanshahi et al., 2015). Here, ascending and descending messages among discrete levels are passed between (Bayesian model) averages of expected hidden states (that have been duplicated in this figure to account for the known laminar specificity of extrinsic cortico-cortical connections). We have placed the outcome prediction errors in deep layers (Layer 5 pyramidal cells) that project to the medium spiny cells of the striatum (Arikuni & Kubota, 1986). These outcome prediction errors are used to compute expected free energy and consequent expectations about policies. Policy expectations are then used to form (Bayesian model) averages of hidden states that are necessary for message passing between discrete hierarchical levels. Similarly, expected outcomes under each policy are passed via corticothalamic connections to the thalamus to evaluate the free energy of each dynamic model, in conjunction with posterior expectations from the continuous level. The resulting posterior expectations are conveyed by thalamocortical projections to inform discrete (hierarchical) message passing. The little arrows referred to the corresponding messages in Figure 9.

Figure 10 sketches an architecture that comprises three levels of a discrete hierarchical model and a single continuous level. In this neural network, we have focused on the extrinsic (between-region) connections that pass ascending prediction errors and expectations about subordinate states—and descending predictions (of initial discrete states or causes of dynamics). In virtue of assigning the sources of ascending messages to superficial pyramidal cells and the sources of descending messages to deep pyramidal cells, this recurrent extrinsic connectivity conforms to known neuroanatomy (Bastos et al., 2012; Felleman and Van Essen, 1991; Markov et al., 2013). An interesting exception here is the laminar specificity of higher-level (discrete) descending projections that arise in deep layers but target state prediction errors assigned to granular layers (as opposed to the more conventional super granular layers). Neuroanatomically, this may reflect the fact that laminar specificity is less pronounced in short-range extrinsic connections (Markov et al., 2013). An alternative perspective rests on the fact that higher (dysgranular) cortical areas often lack a distinct granular layer (Barbas, 2007; Barrett & Simmons, 2015), leading to the speculation that dysgranular cortex may engage in belief updating of categorical or discrete sort.

The belief propagation entailed by policy and action selection in Figure 10 is based upon the anatomy of cortico–basal ganglia–thalamic loops described in Jahanshahi, Obeso, Rothwell, and Obeso (2015). If one subscribes to this functional anatomy, the form of belief propagation suggests that competing low-level (motor executive) policies are evaluated in the putamen; intermediate (associative) policies in the caudate; and high-level (limbic) policies in the ventral striatum. These representations then send (inhibitory or GABAergic) projections to the globus pallidus that encodes the expected (selected) policy. These expectations are then communicated via thalamocortical projections to superficial layers encoding Bayesian model averages. From a neurophysiological perspective, the best candidate for the implicit averaging would be matrix thalamocortical circuits that “appear to be specialized for robust transmission over relatively extended periods, consistent with the sort of persistent activation observed during working memory and potentially applicable to state-dependent regulation of excitability” (Cruikshank et al., 2012, p. 17813). This implicit belief updating is consistent with invasive recordings in primates, which suggest an anteroposterior gradient of time constants (Kiebel, Daunizeau, & Friston, 2008; Murray et al., 2014). Note that the rather crude architecture in Figure 10 does not include continuous (predictive coding) message passing that might operate in lower hierarchical areas of the sensorimotor system. This means that there may be differences in terms of corticothalamic connections in prefrontal regions, compared with primary motor cortex, which has a distinct (agranular) laminar structure. See Shipp et al. (2013) for a more detailed discussion of these regionally specific differences.

The exchange of prior and posterior expectations about discrete outcomes between the categorical and continuous parts of the model have been assigned to corticothalamic loops, while the evaluation of expected free energy and subsequent expectations about policies have been associated with the cortical–basal ganglia–thalamic loops. An interesting aspect of this computational anatomy is that posterior beliefs about where to sample the world next are delivered from higher cortical areas (e.g., parietal cortex), where this salient sampling depends upon subcortical projections, informing empirical prior expectations about where to sample next. One could imagine these arising from the superior colliculus and/or pulvinar in a way that would be consistent with their role as a salience map (Robinson & Petersen, 1992; Veale, Hafed, & Yoshida, 2017). In short, sensory evidence garnered from the continuous level of the model is offered to higher levels in terms of posterior expectations about discrete outcomes. These high levels reciprocate with empirical priors that ensure the right sort of dynamic engagement with the world.

Clearly, there are many anatomical issues that have been ignored here, such as the distinction between direct and indirect pathways (Frank, 2005), the role of dopamine in modulating the precision of beliefs about policies (Friston, Schwartenbeck, et al., 2014), and so on. However, the basic architecture suggested by the above treatment speaks to the biological plausibility of belief propagation under the generative models. This concludes our theoretical treatment of belief propagation in the brain and the implications for intrinsic and extrinsic neuronal circuitry. The following section illustrates the sorts of behavior that emerge under this sort of architecture.

## Simulations of Reading

This section tries to demystify the notions of hidden causes, states, and policies by presenting a simulation of pictographic reading. This simulation is a bit abstract but serves to illustrate the accumulation of evidence over nested timescales—and the integration of discrete categorization with continuous oculomotor sampling of a visual world. Furthermore, it highlights the role of the discrete part of the model in guiding the sampling of a continuous sensorium. We have previously presented the discrete part in the context of scene construction and simulated reading (Mirza et al., 2016). In this paper, we focus on the integration of a Markov decision process model of visual search (Mirza et al., 2016) with a predictive coding model of saccadic eye movements (K. Friston et al., 2012), to produce a complete (mixed) model of evidence accumulation.

**Figure 11:**
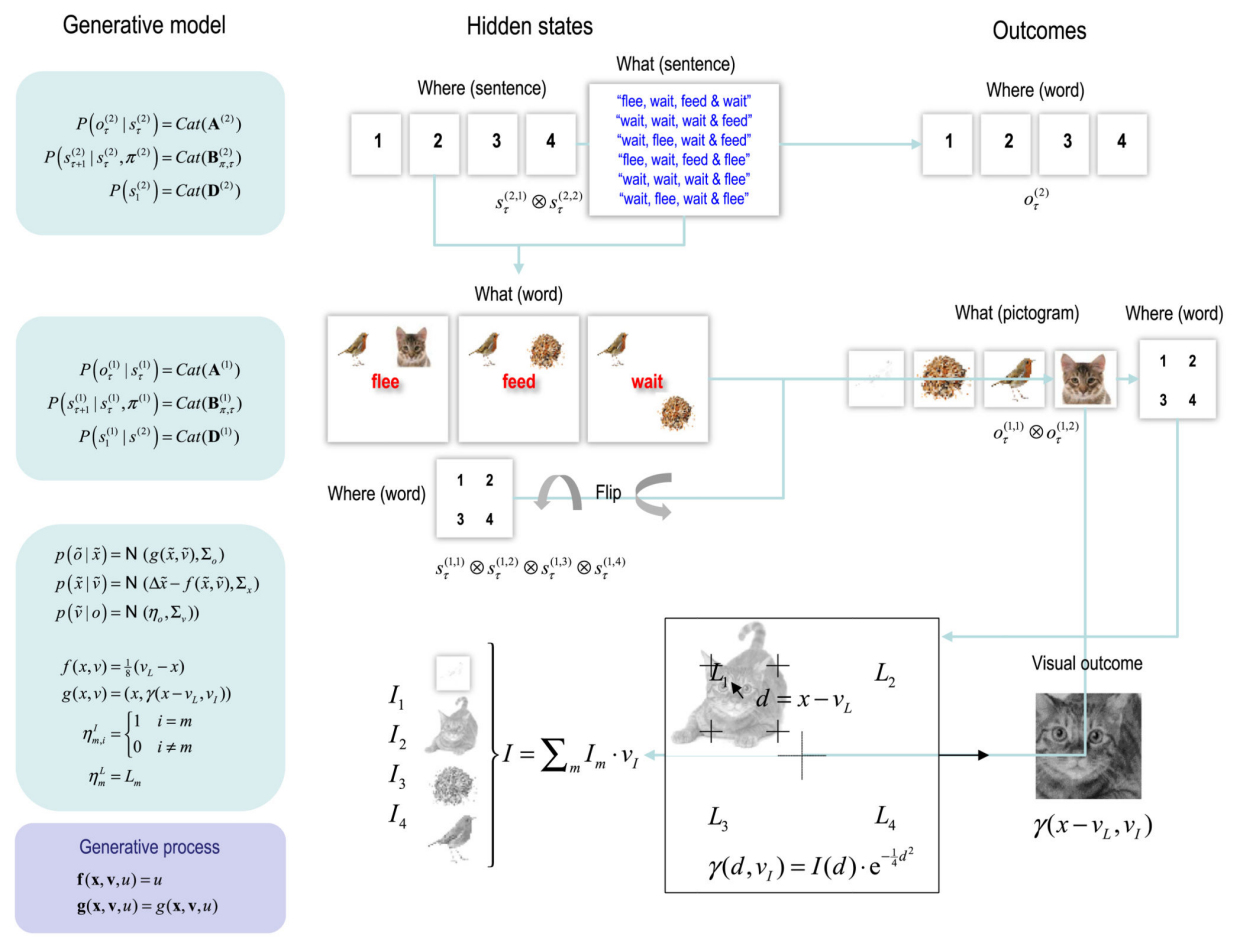
A generative model of pictographic reading. In this model there are two discrete hierarchical levels with two sorts of hidden states at the second level and four at the first level. The hidden states at the higher level correspond to the *sentence* or narrative—generating sequences of *words* at the first level—and which *word* the agent is currently sampling (with six alternative *sentences* and four *words* respectively). These hidden states combine to specify the word at the first level (*flee*, *feed,* or *wait*). The hidden states at the first level comprise the current *word* and which quadrant the agent is looking at. These hidden states combine to generate outcomes in terms of *letters* or pictograms that would be seen at that location*.* In addition, two further hidden states flip the relative locations vertically or horizontally. The vertical flip can be thought of in terms of font substitution (uppercase versus lowercase), while the horizontal flip means a word is invariant under changes to the order of the letters (cf. palindromes). In this example, *flee* means that a bird is next to a cat, *feed* means a bird is next to some seeds, and *wait* means seeds are above (or below) the bird. Notice that there is a (proprioceptive) outcome signaling the *word* currently being sampled (e.g., head position), while at the lower level there are two discrete outcome modalities. The first (exteroceptive) outcome corresponds to the observed *letter* and the second (proprioceptive) outcome specifies a point of visual fixation (e.g., in a head-centered frame of reference). Similarly, there are policies at both levels. The high-level policy determines which word the agent is currently reading, while the lower level dictates eye movements among the quadrants containing letters. These discrete outcomes (the pictogram, *what*, and target location, *where*) generate continuous visuomotor signals as follows: the target location (specified by the discrete *where* outcome) is the center of the corresponding quadrant (denoted by *L* in the figure). This point of fixation attracts the current center of gaze (in the generative model) that is enacted by action (in the generative process), where action simply moves the eye horizontally or vertically. At every point in time, the visual outcome is sampled from an image (with 32 *×* 32 pixels), specified by the discrete *what* outcome. This sampling is eccentric, based upon the displacement between the target location and the current center of gaze (denoted by *d* in the figure). Finally, the image contrast is attenuated as a Gaussian function of displacement to emulate sensory attenuation. In short, the continuous state space model has two hidden causes target location and identity (denoted by *v_L_* and *v_I_*) and a single hidden state (*x*), corresponding to the current center gaze.

In brief, the generative model has two discrete levels. The highest level generates a sentence by sampling from one of six possibilities, where each sentence comprises four words. Given where the (synthetic) subject is currently looking, the sentence therefore specifies a word and the hidden states at the level below. These comprise different letters that can be located in quadrants of the visual field. Given the word and the quadrant currently fixated, the letter is specified uniquely. These hidden states now prescribe the dynamical model; namely, the attracting point of fixation and the content of visual input (i.e., a pictogram) that would be seen at that location. These dynamics are mediated simply by a continuous generative model, in which the center of fixation is attracted to a location prior, while the visual input is determined by the pictogram or letter at that location. Figure 11 provides a more detailed description of the generative model. The discrete parts of this model have been used previously to simulate reading. We will therefore concentrate on the implicit evidence accumulation and prescription of salient target locations for saccadic eye movements. Please see Mirza et al. (2016) for full details of the discrete model.

An interesting aspect of this generative model is that the world or visual scene is represented in terms of affordances; namely, the consequences of acting on—or sampling from—a visual scene. In other words, the generative model does not possess a “sketch pad” on which the objects that constitute a scene (and their spatial or metric relationships) are located. Conversely, the metric aspect of the scene is modeled in terms of “what would happen if I did this.” For example, an object (e.g., letter) is located to the right of another object because “this is the object that I would see if I look to the right.” Note that sensory attenuation ensures that visual impressions are only available after saccadic fixation. This means that the visual stream is composed of a succession of snapshots (that were generated in the following simulations using the same process assumed by the generative model).

To simulate reading, the equations in Figure 10 were integrated using 16 iterations for each time point at each discrete level. At the lowest continuous level, each saccade is assumed to take about 256 ms. (This is roughly the amount of time taken per iteration on a personal computer—to less than an order of magnitude.) This is the approximate frequency of saccadic eye movements, meaning that the simulations covered a few seconds of simulated time.

**Figure 12:**
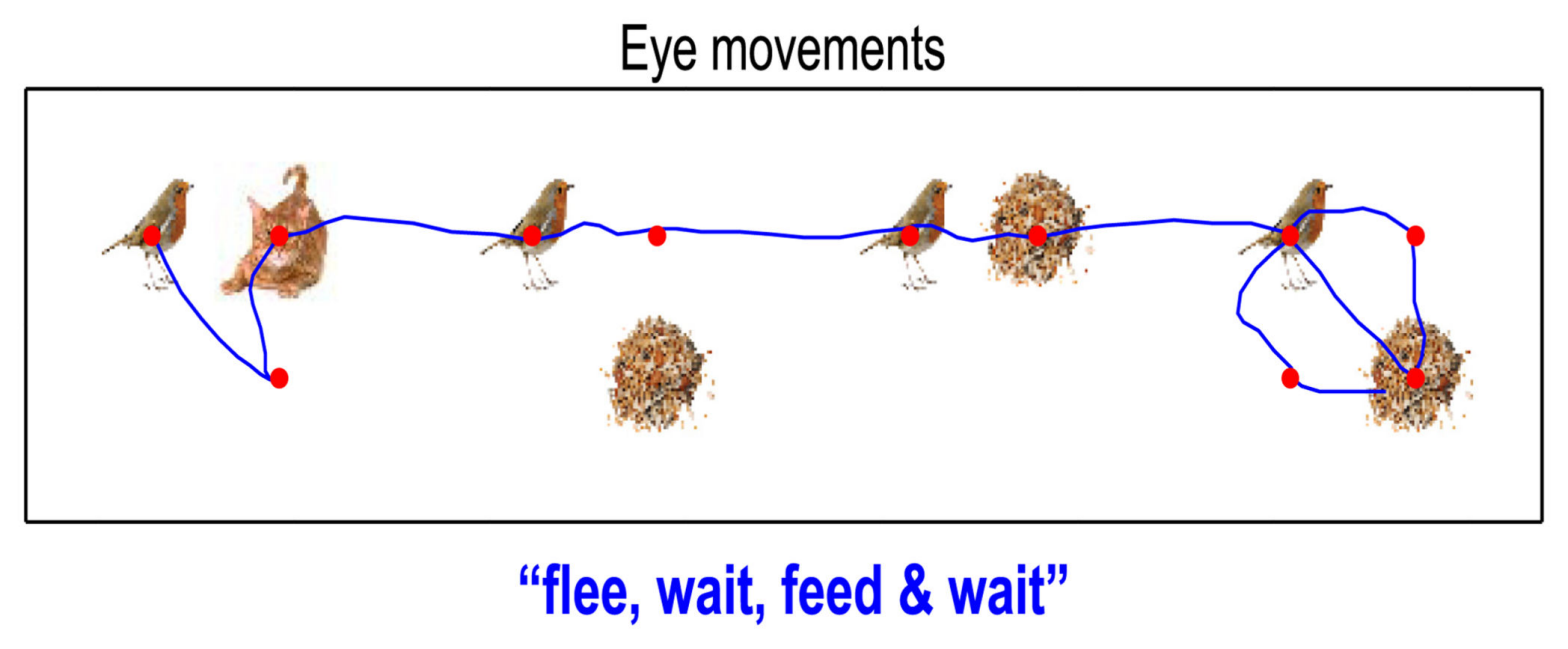
Simulated reading. This figure shows the trajectory of eye movements over four transitions at the second level that entail one or two saccadic eye movements at the first.

Figure 12 shows the behavior that results from integrating the message passing scheme described in Figure 10. Here, we focus on the eye movements within and between the four words (where the generative model assumed random fluctuations with a variance of one eighth). In this example, the subject looks at the first quadrant of the first word and sees a *bird.* She then looks to the lower right and sees nothing, confirming that this word is *flee*—by locating the *cat* on the upper right quadrant. The subject is now fairly confident that the sentence has to be the first or fourth sentence—that both have the same words in the second and third positions. This enables her to quickly scan the subsequent two words (with single saccades to resolve any residual uncertainty) and focus on the last word that disambiguates between two plausible sentences. Here, she discovers *seeds* on the second saccade and empty quadrants off the diagonal. At this point, residual uncertainty about the sentence is resolved and the subject infers the correct sentence.

**Figure 13:**
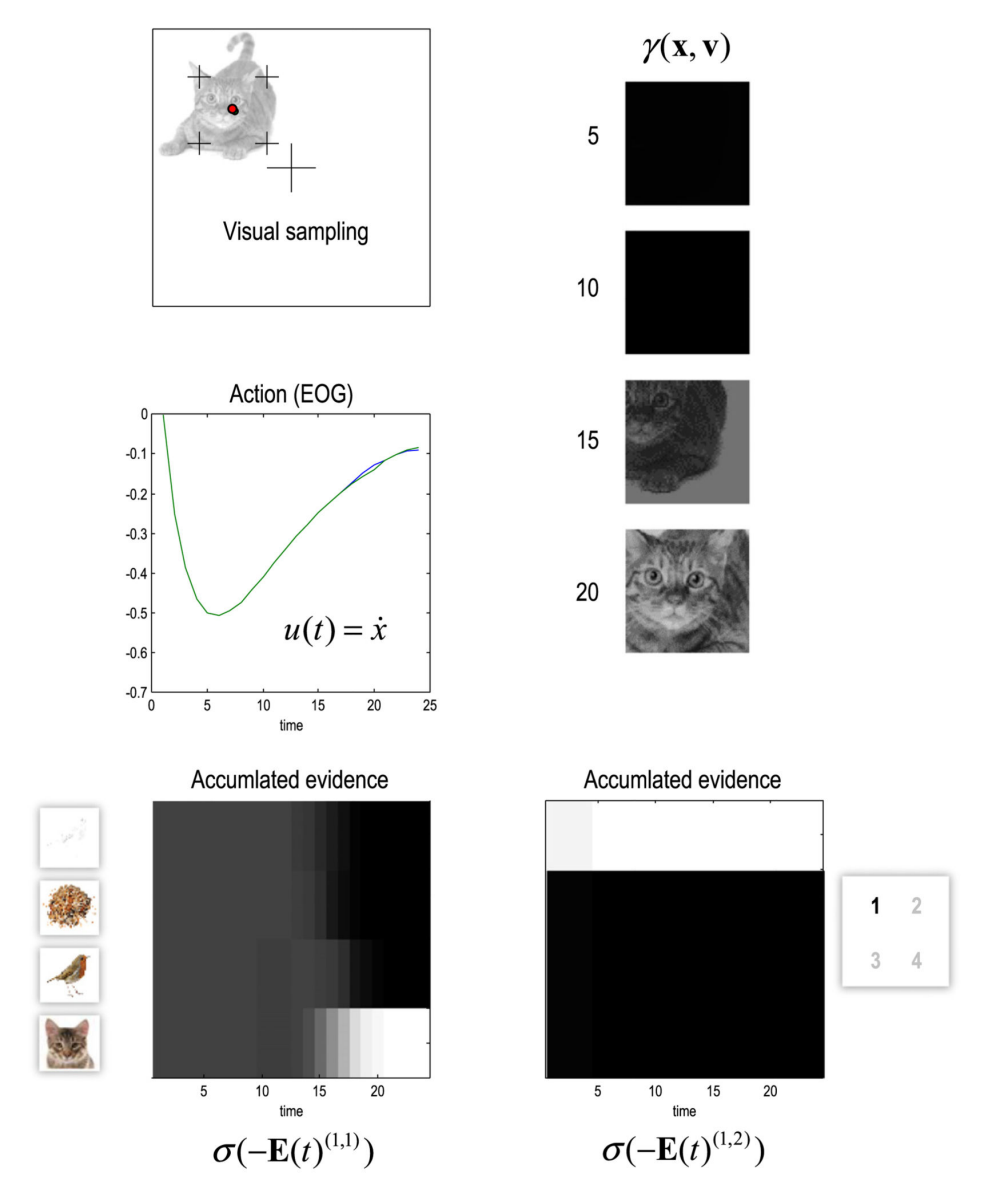
Visual sampling. **Upper left:** Final location of a saccadic eye movement (from the center of the visual field) indicated by a red dot. The green dot is where the subject thought she was looking. The crosshairs demark the field of view. **Middle left:** This plot shows action that corresponds to the motion (in two dimensions) of the hidden state describing the current center of gaze. This trajectory is shown as a function of the time steps used to integrate the generalized belief updating (24 time steps, each corresponding to about 10 ms of real time). **Upper right:** These panels showed the visual samples every five time steps. Note that the luminance contrast increases as the center of gaze approaches the target location. **Lower panels:** These panels illustrate evidence accumulation during the (continuous) saccadic sampling in terms of the posterior probability over (discrete) visual (lower left) and proprioceptive (lower right) outcomes.

An example of the evidence accumulation during a single saccade is provided in Figure 13. In this example, the subject looks from the central fixation to the upper left quadrant to see a cat. The concomitant action is shown as a simulated electroculogram in the middle left panel, with balanced movement in the horizontal and vertical directions. The corresponding visual input is shown for four consecutive points in time (i.e., every five time steps, where each of the 25 time steps of the continuous integration scheme corresponds roughly to 10 ms). Note that the luminance contrast increases as the center of gaze approaches the target location (specified by the empirical prior from the discrete part of the model). This implements a simple form of sensory attenuation. In other words, it precludes precise visual information during eye movement, such that high-contrast information is only available during fixation. Here, the sensory attenuation is implemented by the modulation of the visual contrast as a Gaussian function of the distance between the target and the current center of gaze (see Figure 11). This is quite a crude way of modeling sensory attenuation; however, it is consistent with the fact that we do not experience optic flow during saccadic eye movements.

The lower panels of Figure 13 show the evidence accumulation during the continuous saccadic sampling in terms of the posterior probability under the four alternative visual (*what*) outcomes (lower left) and the four proprioceptive (*where*) outcomes (lower right). In this instance, the implicit model comparison concludes that a *cat* is located in the first quadrant. Note that the posterior probability for the target location is definitive from the onset of the saccade (by virtue of the precise empirical priors from the level above). In contrast, imprecise (empirical prior) beliefs about the visual content take more time to resolve into precise (posterior) beliefs as the location priors are realized via action. This is a nice example of multimodal sensory integration and evidence accumulation. Namely, precise beliefs about the *where* state of the world are used to resolve uncertainty about the *what* aspects in a way that speaks directly to the epistemic affordance that underlies salience.

### Categorization and Evidence Accumulation

**Figure 14:**
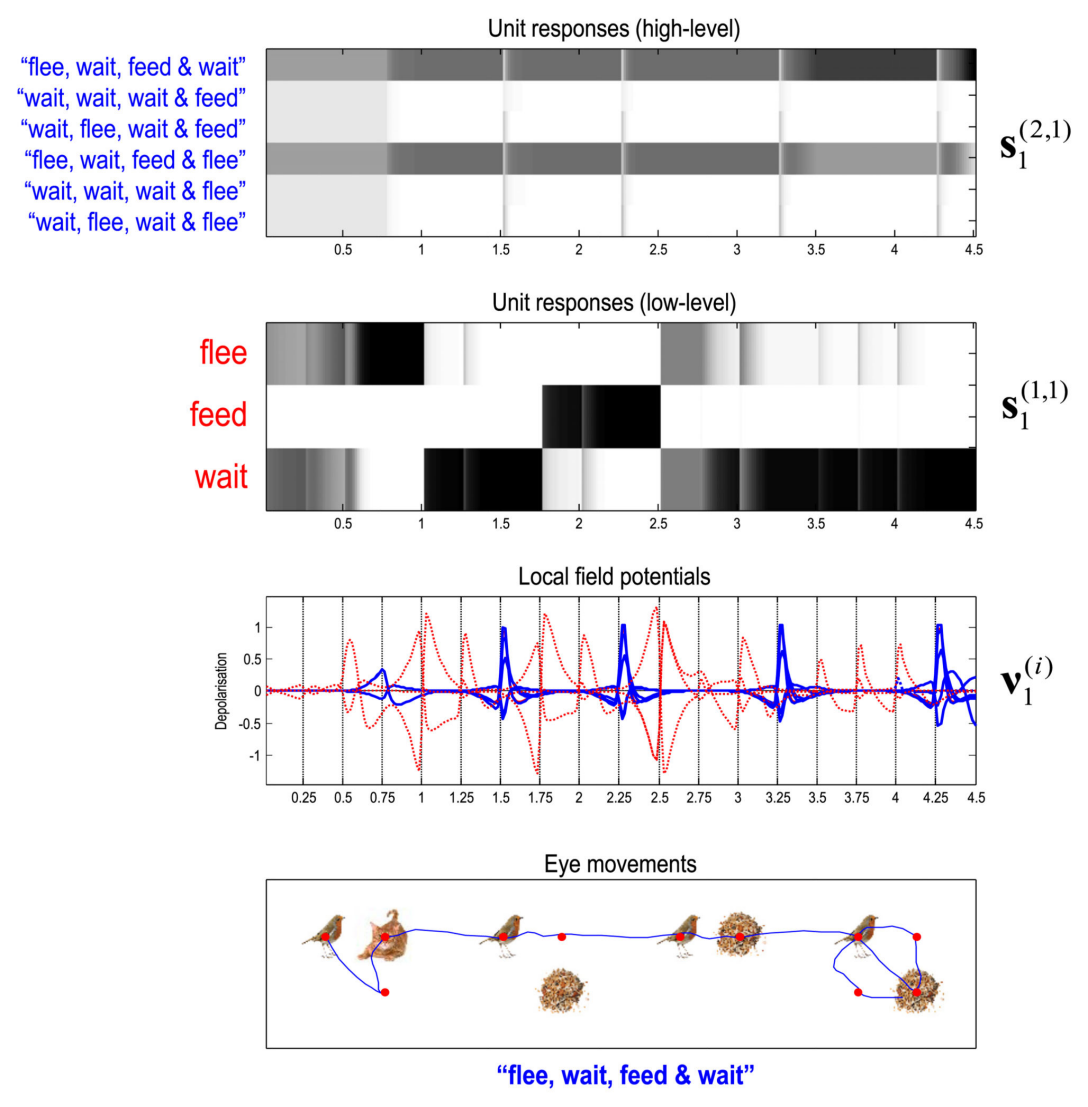
Simulated electrophysiological responses during reading. This figure illustrates belief propagation (producing the behavior shown in Figure 11), in terms of simulated firing rates and depolarization. Expectations about the initial hidden state (at the first time step) at the higher (upper panel) and lower (middle panel) discrete levels are presented in raster format. The horizontal axis is time over the entire simulation, corresponding roughly to 4.5 seconds. Firing rates are shown for populations encoding Bayesian model averages of the six *sentences* at the higher level and the three *words* at the lower level. A firing rate of one corresponds to black. The transients in the third panel are the firing rates in the upper panels filtered between 4 Hz and 32 Hz—and can be regarded as (band-pass filtered) fluctuations in depolarization. Saccade onsets are shown with the vertical (dashed) lines. The lower panel reproduces the eye movement trajectories of Figure 12.

Figure 14 shows the simulated neuronal responses underlying the successive accumulation of evidence at discrete levels of the hierarchical model (cf. Huk & Shadlen, 2005). The neurophysiological interpretations of these results appeal to Equation 6, where expectations are encoded by the firing rates of principal cells and fluctuations in transmembrane potential are driven by prediction errors. A more detailed discussion of how the underlying belief propagation translates into neurophysiology can be found in K. Friston, FitzGerald, et al. (2017).

Under the scheduling used in these simulations, higher-level expectations wait until lower-level updates have terminated and, reciprocally, lower-level updates are suspended until belief updating in the higher level has been completed. This means the expectations are sustained at the higher level, while the lower level gathers information with a saccade or two. The upper two panels show the (Bayesian model) averages of expectations about hidden states encoding the *sentence* (upper panel) and *word* (second panel). The lower two panels show simulated electrophysiological and behavioral responses respectively (reproduced from Figure 12). The key thing to note here is the progressive resolution of uncertainty at both levels—and on different timescales. Posterior expectations about the word fluctuate quickly with each successive visual sample, terminating when the subject is sufficiently confident about what she is sampling. The posterior expectations are then assimilated at the highest level, on a slower timescale, to resolve uncertainty about the sentence in play. Here, the subject correctly infers, on the last saccade of the last word, that the first sentence generated the stimuli. These images or raster plots can be thought of in terms of firing rates in superficial pyramidal populations encoding the Bayesian model averages (see Figure 10). The resulting patterns of firing show a marked resemblance to presaccadic delay period activity in the prefrontal cortex (Funahashi, 2014). The corresponding (simulated) local field potentials are shown below the raster plots. These are just band-pass filtered (between 4 and 32 Hz) versions of the spike rates that can be interpreted in terms of depolarization. The fast and frequent (red) evoked responses correspond to the Bayesian model averages (pertaining to the three possible *words*) at the first level, while the interspersed and less frequent transients (blue) correspond to updates at the highest level (over six *sentences*). The resulting succession of local field potentials or event-related potentials again look remarkably similar to empirical responses in inferotemporal cortex during active vision (Purpura, Kalik, & Schiff, 2003). Although not pursued here, one can perform time frequency analyses on these responses to disclose interesting phenomena such as theta gamma coupling (entailed by the fast updating within saccade that repeats every 250 ms between saccades). In summary, the belief propagation mandated by the computational architecture of the sort shown in Figure 10 leads to a scheduling of message passing that is similar to empirical perisaccadic neuronal responses in terms of both unit activity and event-related potentials.

### Summary

In the previous section, we highlighted the biological plausibility of belief propagation based upon deep temporal models. In this section, this biological plausibility is further endorsed by reproducing electrophysiological phenomena such as perisaccadic delay period firing activity and local field potentials. Furthermore, these simulations have a high degree of face validity in terms of saccadic eye movements during reading (Rayner, 1978, 2009).

## Discussion

We have derived a computational architecture for the brain based upon belief propagation and graphical representations of generative models. This formulation of functional integration offers both a normative and a process theory. It is normative in the sense that there is a clearly defined objective function; namely, variational free energy. An attendant process theory can be derived easily by formulating neuronal dynamics as a gradient descent on this proxy for surprise or (negative) Bayesian model evidence. The ensuing architecture and (neuronal) message passing offers generality at a number of levels. These include deep generative models based on a mixture of categorical states and continuous variables that generate sequences and dynamics. From an algorithmic perspective, we have focused on the link functions or factors that enable categorical representations to talk to representations of continuous quantities, such as position and luminance contrast. One might ask how all this helps us understand the nature of dynamic connectivity in the brain. Clearly, there are an enormous number of anatomical and physiological predictions that follow from the sort of process theory described in this paper; these range from the macroscopic hierarchical organization of cortical areas in the brain to the details of canonical microcircuits (e.g., Adams et al., 2013; Bastos et al., 2012; Friston, 2008; Mumford, 1992; Shipp, 2016). Here, we will focus on two themes: first, the implications for connectivity dynamics within cortical microcircuits and, second, a more abstract consideration of global dynamics in terms of self-organized criticality.

### Intrinsic Connectivity Dynamics

The update equations for both continuous and discrete belief propagation speak immediately to state- or activity-dependent changes in neuronal coupling. Interestingly, both highlight the importance of state-dependent changes in the connectivity of superficial pyramidal cells in supragranular cortical layers. This conclusion rests on the following observations.

Within the belief propagation for continuous states, one can identify the connections that mediate the influence of populations encoding expectations of hidden causes on prediction error units and vice versa. These correspond to connections (*d*) and (6) in Figure 7.

(13) $$\begin{array}{l} {\tilde{\varepsilon }}_{\nu }^{\left(i\right)}={\tilde{\mu }}_{\nu }^{\left(i-1\right)}-\underset{\left(d\right)}{\underset{︸}{g^{\left(i\right)}({\tilde{\mu }}_{x}^{\left(i\right)},{\tilde{\mu }}_{\nu }^{\left(i\right)})}}\\ {\dot{\tilde{\mu }}}_{\nu }^{\left(i\right)}=\Delta {\tilde{\mu }}_{\nu }^{\left(i\right)}+\partial_{\tilde{\nu }}g^{\left(i\right)}\cdot \Pi_{\nu }^{\left(i\right)}{\tilde{\varepsilon }}_{\nu }^{\left(i\right)}+\partial_{\tilde{\nu }}f^{\left(i\right)}\cdot \Pi_{x}^{\left(i\right)}{\tilde{\varepsilon }}_{x}^{\left(i\right)}-\underset{\left(6\right)}{\underset{︸}{\Pi_{\nu }^{\left(i+1\right)}{\tilde{\varepsilon }}_{\nu }^{\left(i+1\right)}}}. \end{array}$$

Biologically, these involve descending extrinsic and intrinsic connections to and within supragranular layers. These connections entail two sorts of context sensitivity. The first arises from the nonlinearity of the functions implicit in the generative model. The second rests on the precision or gain afforded prediction errors. The nonlinearity means that sensitivity of prediction errors (encoded by superficial pyramidal cells) depends upon their presynaptic input mediating top-down predictions, rendering the coupling activity dependent. The changes in coupling due to precision have not been discussed in this paper; however, there is a large literature associating the optimization of precision with sensory attenuation and attention (Auksztulewicz & Friston, 2015; Bauer, Stenner, Friston, & Dolan, 2014; Brown, Adams, Parees, Edwards, & Friston, 2013; Kanai, Komura, Shipp, & Friston, 2015; Pinotsis et al., 2014); namely, a precision engineered gain control of prediction error units (i.e., superficial pyramidal cells). The mediation of this gain control is a fascinating area that may call upon classical neuromodulatory transmitter systems or population dynamics and the modulation of synchronous gain (Aertsen et al., 1989). This (population dynamics) mechanism may be particularly important in understanding attention in terms of communication through coherence (Akam & Kullman, 2012; Fries, 2005) and the important role of inhibitory interneurons in mediating synchronous gain control (Kann, Papageorgiou, & Draguhn, 2014; Lee, Whittington, & Kopell, 2013; Sohal, Zhang, Yizhar, & Deisseroth, 2009). In short, much of the interesting context sensitivity that leads to dynamic connectivity can be localized to the excitability of superficial pyramidal cells. Exactly the same conclusion emerges when we consider the update equations for categorical states.

Figure 2 suggests that the key modulation of intrinsic (cortical) connectivity is mediated by policy expectations. These implement the Bayesian model averaging (over policy-specific estimates of states) during state estimation. Physiologically, this means that the coupling between policy-specific states (here assigned to supragranular interneurons) and the Bayesian model averages (here assigned to superficial pyramidal cells) is a key locus of dynamic connectivity. This suggests that fluctuations in the excitability of superficial pyramidal cells are a hallmark of belief propagation under the discrete process theory on offer.

These conclusions are potentially important from the point of view of empirical studies. For example, it suggests that dynamic causal modeling of condition-specific, context-sensitive, effective connectivity should focus on intrinsic connectivity; particularly, connections involving superficial pyramidal cells that are the source of forward extrinsic (between region) connections in the brain (e.g., Auksztulewicz & Friston, 2015; Brown & Friston, 2012; Fogelson, Litvak, Peled, Fernandez-del-Olmo, & Friston, 2014; Pinotsis et al., 2014). Indeed, several large-scale neuronal simulations speak to the potential importance of intrinsic excitability (or excitation-inhibition balance) in setting the tone for—and modulating—cortical interactions (Gilson, Moreno-Bote, Ponce-Alvarez, Ritter, & Deco, 2016; Roy et al., 2014).

Clearly, a focus on intrinsic excitability is important from a neurophysiological and pharmacological perspective. This follows from the fact that the postsynaptic gain or excitability of superficial pyramidal cells depends upon many neuromodulatory mechanisms. These include synchronous gain (Chawla, Lumer, & Friston, 1999) that is thought to be mediated by interactions with inhibitory interneurons that are, themselves, replete with voltage-sensitive NMDA receptors (Lee et al., 2013; Sohal et al., 2009). Not only are these mechanisms heavily implicated in things like attentional modulation (Fries, Reynolds, Rorie, & Desimone, 2001), they are also targeted by most psychotropic drugs and (endogenous) ascending modulatory neurotransmitter systems (Dayan, 2012). This focus—afforded by computational considerations—deals with a particular aspect of microcircuitry and neuromodulation. Can we say anything about whole-brain dynamics?

### Self-Evidencing and Self-Organized Criticality

Casting neuronal dynamics as deterministic belief propagation may seem to preclude characterizations that appeal to dynamical systems theory (Baker et al., 2014; Breakspear, 2004); in particular, notions like metastability, itinerancy, and self-organized criticality (Bak, Tang, & Wiesenfeld, 1987; Breakspear, 2004; Breakspear & Stam, 2005; Deco & Jirsa, 2012; Jirsa, Friedrich, Haken, & Kelso, 1994; Kelso, 1995; Kitzbichler, Smith, Christensen, & Bullmore, 2009; Shin & Kim, 2006; Tsuda & Fujii, 2004). However, there is a deep connection between these phenomena and the process theory evinced by belief propagation. This rests upon the minimization of variational free energy in terms of neuronal activity encoding expected states. For example, from Equation 8, we have the following:

(14) $$\begin{array}{c} {\dot{\tilde{\mu }}}_{x}=\Delta {\tilde{\mu }}_{x}-\partial_{\tilde{x}}F\\ \;=-(\partial_{\tilde{x}\tilde{x}}F-\Delta ){\tilde{\mu }}_{x} \end{array}.$$

On the dynamical system view, the curvature of the free energy plays the role of a Jacobian, whose eigenvalues *λ* = *eig*(𝜕_*x̃x̃*_*F* − Δ) = *eig*(𝜕_*x̃x̃*_*F*) > 0 determine the dynamical stability of belief propagation (note that Δ does not affect the eigenvalues because the associated flow does not change free energy). Technically, the averages of these eigenvalues are called *Lyapunov exponents*, which characterize deterministic chaos. In brief, small eigenvalues imply a slower decay of patterns of neuronal activity (that correspond to the eigenvectors of the Jacobian). This means that a small curvature necessarily entails a degree of dynamical instability and *critical slowing*; that is, it takes a long time for the system to recover from perturbations induced by, for example, sensory input.

The key observation here is that the curvature of free energy is necessarily small when free energy is minimized. This follows from the fact that (under the Laplace assumption of a Gaussian posterior) the entropy part of variational free energy, *H*, becomes the (negative) curvature of the energy, *U*:

(15) $$\begin{array}{l} \;F=U-H\\ \;H=-\frac{1}{2}\text{ln}|\partial_{\tilde{x}\tilde{x}}U|\\ \;U=-\text{ln}\;p(\tilde{o},\tilde{x}={\tilde{\mu }}_{x})\\ \begin{array}{l} \partial_{\tilde{x}\tilde{x}}F\approx \partial_{\tilde{x}\tilde{x}}U\Rightarrow \end{array}\\ \;F\approx U+\frac{1}{2}\text{ln}|\partial_{xx}F|\\ \;=U+\frac{1}{2}\sum_{i}\text{ln}\lambda_{i} \end{array}.$$

This means that free energy can be expressed in terms of the energy plus the log eigenvalues or *Lyapunov exponents*. The remarkable thing here is that because belief propagation is trying to minimize variational free energy *it is also trying to minimize the Lyapunov exponents*, which characterize the dynamics. In other words, belief propagation organizes itself towards critical slowing. This formulation suggests something quite interesting. Self-organized criticality and unstable dynamics are a necessary and emergent property of belief propagation. In short, if one subscribes to the free energy principle, self-organized criticality is an epiphenomenon of the underlying imperative with which all self-organizing systems must comply; namely, to minimize free energy or maximize Bayesian model evidence. From the perspective of active inference, this implies self-evidencing (Hohwy, 2016), which entails self-organized criticality (Bak et al., 1987) and dynamic connectivity (Allen et al., 2012; Breakspear, 2004). In this view, the challenge is to impute the hierarchical generative models—and their message passing schemes—that best account for functional integration in the brain. Please see K. J. Friston, Kahan, Razi, Stephan, and Sporns (2014) for a fuller discussion of this explanation for self-organized criticality, in the context of effective connectivity and neuroimaging.

## Supporting Information

Supporting Information: http://www.fil.ion.ucl.ac.uk/spm/

Although the generative model changes from application to application, the belief updates described in this paper are generic and can be implemented using standard routines (here **spm_MDP_VB_X.m** and **spm_ADEM.m**). These routines are available as Matlab code in the SPM academic software: http://www.fil.ion.ucl.ac.uk/spm/software. The simulations in this paper can be reproduced (and customized) via a graphical user interface by typing in >> **DEM** and selecting the **Mixed models** demo.

Appendices and Tables

## Footnotes

Generative model: Or forward model; a probabilistic mapping from causes to observed consequences (data). It is usually specified in terms of the likelihood of getting some data given their causes (parameters of a model) and priors on the parameters.

Generalized coordinates of motion: Cover the value of a variable, its motion, acceleration, jerk, and higher orders of motion. A point in generalized coordinates corresponds to a path or trajectory over time.

Factor graph: A bipartite graph (where two distinct sets of nodes are connected by edges) representing the factorization of a function, usually a probability distribution function. Formulating a Bayesian network or model as a factor graph enables the efficient computation of marginal distributions, through the sum-product algorithm.

Bayesian network: A Bayes network, model, or belief network is a probabilistic graphical model that represents a set of random variables and their conditional dependencies via a directed acyclic graph.

Free energy: An information theory measure that bounds (is greater than) the surprise on sampling some data, given a generative model.

Empirical prior: Priors that are induced by hierarchical models; they provide constraints on the recognition density in the usual way but depend on the data.

Surprise: Surprisal or self-information is the negative log probability of an outcome. An improbable outcome is therefore surprising.

Prior: The probability distribution or density on the causes of data that encode beliefs about those causes prior to observing the data.

(Kullback-Leibler) Divergence: Information divergence, information gain, or relative entropy is a noncommutative measure of the difference between two probability distributions.

Gradient descent: An optimization scheme that finds a minimum of a function by changing its arguments in proportion to the negative of the gradient of the function at the current value.

Precision: In general statistical usage, the inverse variance or dispersion of a random variable. The precision matrix of several variables is also called a concentration matrix. It quantifies the degree of certainty about the variables.
