# Supplementary material for: The graphical brain: Belief propagation and active inference
Source: Netw Neurosci. 2017 Dec 1;1(4):381–414. doi: 10.1162/NETN_a_00018 (PMC5798592; doi:10.1162/NETN_a_00018)
Supplement: Supplementary file 1 [file netn-01-381-s001.pdf]

Friston, K. J., Parr, T., & de Vries, B. (2017). Appendices for "The graphical brain: Belief propagation and active inference." *Network Neuroscience, 1*(4), 381−414. https://doi.org/10.1162/netn_a_00018

Appendices

Appendix 1 – *Expected free energy*: variational free energy is a functional of an approximate posterior distribution over states $Q(s)$, given observed outcomes *o*, under a probabilistic generative model $P(o,s)$:

$$\begin{aligned} F &= E_Q[\ln Q(s) - \ln P(o,s)] \\ &= D[Q(s) \,||\, P(s)] - E_Q[\ln P(o \mid s)] \end{aligned} \quad \text{(A.1}$$

The second equality expresses free energy as the difference between a Kullback-Leibler divergence (i.e., *complexity*) and the expected log likelihood (i.e., *accuracy*), given (observed) outcomes.

In contrast, the expected free energy is a functional of the probability over hidden states *and* (unobserved) outcomes, given some policy π that determines the distribution over states. This can be expressed as:

$$\begin{aligned} G &= E[\ln P(s \mid \pi) - \ln P(o,s \mid \pi)] \\ &= E[\ln P(s \mid \pi) - \ln P(o \mid s) - \ln P(s \mid \pi)] \\ &= E[H[P(o \mid s)]] \end{aligned} \quad \text{(A.2}$$

The expected free energy is therefore just the expected entropy (i.e. *uncertainty*) about outcomes under a particular policy. Things get more interesting if we express the generative model in terms of a prior over outcomes that does not depend upon the policy

$$\begin{aligned} G &= E[\ln P(s \mid \pi) - \ln P(s \mid o,\pi)] - E[\ln P(o)] \\ &= D[P(o \mid \pi) \,||\, P(o)] + E[H[P(o \mid s)]] \end{aligned} \quad \text{(A.3}$$

The first equality expresses expected free energy in terms of (negative) *intrinsic* and *extrinsic value*, while the second expression shows an equivalent formulation in terms of the relative uncertainty about outcomes given prior beliefs (i.e., *risk*) and expected uncertainty about outcomes, given their causes (i.e., *ambiguity*). This is the form used in active inference, where the probabilities in (A.3) are conditioned upon past observations (such that the generative model P is replaced by an approximate posterior Q): see (A.5).

Appendix 2 – *Belief updating*: approximate Bayesian inference corresponds to minimising marginal or variational free energy, with respect to sufficient statistics that constitute posterior beliefs. For generative models of discrete states, free energy can be expressed as the (time-dependent) free energy under each policy plus the complexity incurred by posterior beliefs about (time-invariant) policies, where (with some simplifications):

$$\begin{aligned} F &= D[Q(s_1,\dots,s_T \mid \pi)Q(\pi) \,\|\, P(s_1,\dots,s_T,\pi)] - \mathrm{E}_Q[\ln P(o_1,\dots,o_\tau \mid s_1,\dots,s_T)] \\ &= \textstyle\sum_\tau \mathrm{E}_Q[F(\pi,\tau)] + D[Q(\pi) \,\|\, P(\pi)] \\ &= \boldsymbol{\pi} \cdot (\mathbf{F} + \ln \boldsymbol{\pi} + \mathbf{G}) \end{aligned}$$

The free energy of hidden states is then given by:

$$\begin{aligned} \mathbf{F}_\pi &= \textstyle\sum_\tau F(\pi,\tau) \\ F(\pi,\tau) &= \underbrace{D[Q(s_\tau \mid \pi) \,\|\, P(s_\tau \mid s_{\tau-1},\pi)]}_{complexity} - \underbrace{E[\ln P(o_\tau \mid s_\tau)]}_{accuracy} \\ &= \mathbf{s}_{\pi,\tau} \cdot (\ln \mathbf{s}_{\pi,\tau} - \ln \mathbf{B}_{\pi,\tau-1}\mathbf{s}_{\pi,\tau-1} - \ln \mathbf{A} \cdot o_\tau) \end{aligned} \qquad \text{(A.4}$$

The expected free energy of a policy has a similar form but the expectation is over hidden states and outcomes that have yet to be observed (c.f., Equation A.3); namely, $Q(o_\tau, s_\tau) = P(o_\tau \mid s_\tau) Q(s_\tau \mid \pi)$.

$$
\begin{aligned}
\mathbf{G}_\pi &= \sum\nolimits_\tau G(\pi, \tau) \\
G(\pi, \tau) &= -\underbrace{E[\ln Q(s_\tau \mid o_\tau, \pi) - \ln Q(s_\tau \mid \pi)]}_{intrinsic\ value} - \underbrace{E[\ln Q(o_\tau)]}_{extrinsic\ value} \\
&= \underbrace{D[Q(o_\tau \mid \pi) \,||\, Q(o_\tau)]}_{risk} + \underbrace{E[H[Q(o_\tau \mid s_\tau)]]}_{ambiguity} \\
&= \mathbf{o}_{\pi,\tau} \cdot (\ln \mathbf{o}_{\pi,\tau} + \mathbf{C}_\tau) + \mathbf{H} \cdot \mathbf{s}_{\pi,\tau}
\end{aligned}
$$

$$
\begin{aligned}
\mathbf{H} &= -diag(\mathbf{A} \cdot \ln \mathbf{A}) \\
Q(o_\tau) &= \sigma(-\mathbf{C}_\tau)
\end{aligned}
\qquad \text{(A.5}
$$

Note that the free energy *per se* does not appear in the update equations in this paper. This is because we only consider policies that comprise past actions and one subsequent (allowable) action. This means that the free energies of all policies are the same.

Table 1a – Expressions pertaining to models of discrete states: the shaded rows describe hidden states and auxiliary variables, while the remaining rows describe model parameters and functionals.

Expression	Description
$o_\tau \in \{0,1\}$<br>$\mathbf{o}_\tau = \sum_\pi \boldsymbol{\pi}_\pi \cdot \mathbf{o}_{\pi,\tau} \in [0,1]$	Outcomes<br>and their posterior expectations
$\mathbf{o}_{\pi,\tau} = \mathbf{A}\mathbf{s}_{\pi,\tau}$	Expected outcome, under a particular policy
$s_\tau \in \{0,1\}$<br>$\mathbf{s}_\tau = \sum_\pi \boldsymbol{\pi}_\pi \cdot \mathbf{s}_{\pi,\tau} \in [0,1]$	Hidden states<br>and their posterior expectations
$\pi \in \{1,\ldots,K\}$<br>$\boldsymbol{\pi} = (\boldsymbol{\pi}_1,\ldots,\boldsymbol{\pi}_K) \in [0,1]$	Policies specifying action sequences<br>and their posterior expectations
$u \in \{1,\ldots,U\}$<br>$U_{\pi,\tau} \in \{1,\ldots,U\}$	Allowable actions<br>and sequences of actions under each policy
$\mathbf{v}_{\pi,\tau} = \ln_{\pi,\tau}$<br>$\mathbf{s}_{\pi,\tau} = \sigma(\mathbf{v}_{\pi,\tau})$	Auxiliary variable representing depolarisation<br>and expected state, under a particular policy
$\boldsymbol{\varepsilon}_{\pi,\tau}$<br>$\varsigma_{\pi,\tau}$	Auxiliary variables representing state prediction error<br>and outcome prediction error
$\mathbf{A}$	The likelihood of an outcome under each hidden state
$\mathbf{B}_{\pi,\tau} \in \{\mathbf{B}_1,\ldots\mathbf{B}_U\}$<br>$\mathbf{B}_{\pi,\tau} \square \mathbf{B}_{U_{\pi,\tau}}$	Transition probability for hidden states under each action that is uniquely specified by the policy and time
$\mathbf{C}_\tau = -\ln P(o_\tau)$	Prior surprise about outcomes; i.e., prior cost or inverse preference
$\mathbf{D}$	Prior expectations about initial hidden states
$\mathbf{F}_\pi = \sum_\tau F(\pi,\tau)$	Variational free energy for each policy

$\mathbf{G}_\pi = \sum_\tau G(\pi,\tau)$	Expected free energy for each policy
$\mathbf{H} = -diag(\mathbf{A} \cdot \ln \mathbf{A})$	Entropy or ambiguity about outcomes under each state
$\sigma(-\mathbf{G})_\pi = \dfrac{\exp(-\mathbf{G}_\pi)}{\sum_\pi \exp(-\mathbf{G}_\pi)}$	Softmax function, returning a vector that constitutes a proper probability distribution.

Table 1b – Expressions pertaining to models of continuous states: the shaded rows describe hidden states and auxiliary variables, while the remaining rows describe model and link functions.

Expression	Description
$\tilde{o}(t) = (o, o', o'', \ldots) \in \square$	Outcomes in generalised coordinates of motion
$\tilde{x}(t) = (x, x', x'', \ldots) \in \square$	Hidden states in generalised coordinates of motion
$\tilde{v}(t) = (v, v', v'', \ldots) \in \square$	Hidden causes in generalised coordinates of motion
$u(t)$	Control states or action variables
$\tilde{\varepsilon}_x$<br>$\tilde{\varepsilon}_v$	Auxiliary variables encoding state prediction errors and prediction error on causes
$\tilde{\mu}_x$<br>$\tilde{\mu}_v$	Expected states<br>and causes in generalised coordinates of motion
$f(\tilde{x}, \tilde{v})$<br>$g(\tilde{x}, \tilde{v})$	Equations of motion<br>and nonlinear mapping in the generative model
$\mathbf{f}(\tilde{\mathbf{x}}, \tilde{\mathbf{v}}, u)$<br>$\mathbf{g}(\tilde{\mathbf{x}}, \tilde{\mathbf{v}}, u)$	Equations of motion<br>and nonlinear mapping in the generative process
$\eta = \sum_m \eta_m \cdot \mathbf{o}_{\tau,m}$	Priors over hidden causes (Bayesian model average)

$\mathbf{r}_\tau = \sigma(-\mathbf{F})$	Posterior expectation of discrete outcomes (Bayesian model comparison)
$\mathbf{E}_m = -\ln \mathbf{o}_{\tau,m} - \mathbf{L}_m$	Free energy of the *m*-th discrete outcome
$\mathbf{L}_m = L(\eta_m, \eta, \tilde{\mu}_v)$	Log likelihood of the *m*-th discrete outcome
∂<br>Δ	Functional partial derivative<br>and (matrix) derivative operator on generalised states
